# MALDI Imaging Mass Spectrometry for *In Situ* Proteomic Analysis of Preneoplastic Lesions in Pancreatic Cancer

**DOI:** 10.1371/journal.pone.0039424

**Published:** 2012-06-26

**Authors:** Barbara M. Grüner, Hannes Hahne, Pawel K. Mazur, Marija Trajkovic-Arsic, Stefan Maier, Irene Esposito, Evdokia Kalideris, Christoph W. Michalski, Jörg Kleeff, Sandra Rauser, Roland M. Schmid, Bernhard Küster, Axel Walch, Jens T. Siveke

**Affiliations:** 1 II. Medizinische Klinik, Technische Universität München, Munich, Germany; 2 Chair of Proteomics and Bioanalytics, Center of Life and Food Sciences, Technische Universität München, Munich, Germany; 3 Institute of Pathology, Helmholtz Center Munich - German Research Center for Environmental Health, Neuherberg, Germany; 4 Institute of Pathology, Technische Universität München, Munich, Germany; 5 Department of Surgery, Technische Universität München, Munich, Germany; 6 Center for Integrated Protein Science Munich, Munich, Germany; University of Munich, Germany

## Abstract

The identification of new biomarkers for preneoplastic pancreatic lesions (PanINs, IPMNs) and early pancreatic ductal adenocarcinoma (PDAC) is crucial due to the diseasés high mortality rate upon late detection. To address this task we used the novel technique of matrix-assisted laser desorption/ionization (MALDI) imaging mass spectrometry (IMS) on genetically engineered mouse models (GEM) of pancreatic cancer. Various GEM were analyzed with MALDI IMS to investigate the peptide/protein-expression pattern of precursor lesions in comparison to normal pancreas and PDAC with cellular resolution. Statistical analysis revealed several discriminative *m/z*-species between normal and diseased tissue. Intraepithelial neoplasia (PanIN) and intraductal papillary mucinous neoplasm (IPMN) could be distinguished from normal pancreatic tissue and PDAC by 26 significant *m/z*-species. Among these *m/z*-species, we identified Albumin and Thymosin-beta 4 by liquid chromatography and tandem mass spectrometry (LC-MS/MS), which were further validated by immunohistochemistry, western blot, quantitative RT-PCR and ELISA in both murine and human tissue. Thymosin-beta 4 was found significantly increased in sera of mice with PanIN lesions. Upregulated PanIN expression of Albumin was accompanied by increased expression of liver-restricted genes suggesting a hepatic transdifferentiation program of preneoplastic cells. In conclusion we show that GEM of endogenous PDAC are a suitable model system for MALDI-IMS and subsequent LC-MS/MS analysis, allowing *in situ* analysis of small precursor lesions and identification of differentially expressed peptides and proteins.

## Introduction

Pancreatic ductal adenocarcinoma (PDAC) is the fourth leading cause of cancer death in the western world [1]. Due to the advanced stage at diagnosis and the high intrinsic resistance to therapy, the incidence of PDAC corresponds with its mortality with a median survival of less than 6 month and an overall 5-year-survival rate below 5% [2]. Identification of proteins expressed in preneoplastic lesions may help identify the disease in a preinvasive state, a clinically highly relevant goal as resection remains the only curative approach often frustrated by early undetected metastasis or locally advanced disease [2].

Clinical and histopathological studies have identified three PDAC precursor lesions: pancreatic intraepithelial neoplasia (PanIN), intraductal papillary mucinous neoplasm (IPMN) and mucinous cystic neoplasm (MCN). The by far most common precursors are PanIN lesions, although due to improved imaging modalities cystic neoplasms such as IPMNs and, to a lesser extent, MCNs are increasingly diagnosed [3], [4]. The identification and classification of PanINs as precursors of PDAC [5] has enabled the development of a morphological and genetic progression model (overview in [3]). These advances have contributed to the development of sophisticated *Cre/lox*-based genetically engineered mice (GEM) for endogenous PDAC [6]. A well-established mouse model recapitulating the molecular and morphological stages of human PDAC development is the *Kras^G12D^* model, in which oncogenic *Kras^G12D^* is activated in the endogenous *Kras* locus. Mice develop locally invasive and metastatic PDAC through defined PanIN lesions progressing from PanIN1 to PanIN3 [7]. Additional activation of EGFR signaling leads to an accelerated development of PDAC through PanIN and IPMN lesions, extending the spectrum of clinically relevant PDAC mouse models [8]. Because of the defined genetic background and the experimentally addressable time course of preneoplastic lesion development and progression to PDAC, we hypothesized these models to be valuable study tools for establishing a preclinical early detection biomarker identification approach.

Matrix-assisted laser desorption/ionization (MALDI) imaging mass spectrometry (IMS) has evolved as a novel technique and promising tool in biomarker discovery and translational oncology [9] . Examples include Parkinsońs [10] and Alzheimer’s [11] disease as well as several cancers including gliomas [12], [13], [14], [15], ovarian [16], prostate [17], breast [18] and colon cancer [19]. By providing a molecular *ex vivo* view of the resected tissue, the label-free tracking of endogenous compounds with spatial resolution and molecular specificity is enabled (overview in [9], [20]).

In this study, we applied MALDI-IMS to examine the feasibility of this technique for the identification of potential novel biomarkers in PanIN lesions. We characterized two PanIN-specific peaks, which were identified as ALB1 and TMSB4X. We further substantiate ALB1 expression as part of a hepatic transdifferentiation program of precursor lesions and provide evidence for increased serum levels of TMSB4X in mice with PanIN lesions.

## Materials and Methods

### Ethics Statement

The study was approved by the Ethics committee of the Faculty of Medicine of the Technical University of Munich. Written informed consent was obtained from all patients prior to inclusion in the study.

All animal experiments were conducted in accordance with German Federal Animal Protection Laws and approved by the Institutional Animal Care and Use Committee at the Technical University of Munich.

### Mouse Strains

*Kras^+/LSL−G12D^, Ptf1a^+/Cre^, Ela-Tgfa* and *Trp53^+/LSL−R172H^* strains have been described previously [7], [8], [21], [22], [23]. Mice were interbred to obtain the mouse lines *Ptf1a^+/Cre^;Kras^+/G12D^;Ela-Tgfα; Ptf1a^+/Cre^;Kras^+/G12D^* and *Ptf1a^+/Cre^;Kras^+/G12D^;Ela-Tgfα;Trp53^+/LSL−R172H^* and were backcrossed to C57BL/6J background for at least four generations. C57BL/6J mice served as control.

### Human Samples

Serum samples were obtained from 57 subjects with a histologically proven diagnosis of pancreatic ductal adenocarcinoma (21 women, 26 men, median age 67.1 years). Whole blood was collected prior to surgery. Control serum samples were taken from 10 healthy subjects (2 women, 8 men, median age 66.2 years) and from 12 patients with chronic pancreatitis (3 women, 9 men, median age 55.8 years).

### MALDI-IMS on Tissue Sections from Mouse Pancreata

For MALDI-IMS pancreata were resected and snap-frozen in liquid nitrogen without any pretreatment. 10 µm cryosections were cut and transferred to Indium-Tin-Oxide (ITO) coated glass slides pretreated with poly-lysine 1∶1 in water with 0.1% NP-40. Sections were fixed in 70% ethanol and 100% ethanol for one min. Matrix (10 g/l sinapinic acid in 60% acetonitrile and 0.2% trifluoroacetic acid) was uniformly deposited on the slide using the ImagePrep device (Bruker Daltonics). Mass spectra were measured using the MALDI TOF/TOF Analyzer Ultraflex III (Bruker Daltonics) with a spatial resolution of 70 µm in linear mode. Ions were detected in a mass range of *m/z* 2500 to 25000 with a sampling rate of 0.1 GS/s. A ready-made protein standard (Bruker Daltonics) was employed for calibration of spectra, which was done externally on the same target before each measurement. After measurement the slides were washed in 70% ethanol to remove the matrix and counterstained with hematoxylin/eosin (H&E). High-resolution images of stained sections were taken using the Mirax Scan system (Carl Zeiss) and co-registered with the MALDI-IMS data to correlate mass spectra with the histological features of the same section.

### Statistical Analysis of MALDI-IMS Data

MALDI-IMS data were obtained and analyzed using the FlexControl 3.0, FlexImaging 3.0 and the ClinProTools 2.2 software (Bruker). With the FlexImaging software regions of interest (ROI) were defined according to the morphology (PanIN, IPMN, PDAC, WT) and 40 randomly chosen single spectra per mouse per ROI-group were exported to ClinProTools for further analysis. Respective lesions were classified by an expert pancreatic pathologist (I.E.). The extracted mass spectra were recalibrated on common “background” peaks (spectral alignment) and normalized on their total ion count. In all analyses, the spectra of two groups of ROIs were compared and *p* values were calculated with the combined Wilcoxon rank-sum test for two non-parametric, ordinal, independent samples and Benjamini-Hochberg corrected. *P* values ≤0.05 were considered significant.

For validation of discriminating peaks the Significance Analysis of Microarrays (SAM) test was performed and features with a false discovery rate less than 0.001 were considered significant. The optimal discriminating threshold was determined using Receiver Operating Characteristics (ROC) analysis. Validation was performed with an independent set of samples (Fisher exact t-test, *p*<0.001).

### Peptide and Protein Identification by Liquid Chromatography and Tandem Mass Spectrometry (LC-MS/MS)

Peptides and proteins were extracted directly from sinapinic acid prepared tissue sections. For the extraction, 1 µl of 30% acetonitrile in 0.1% trifluoroacetic acid was applied onto the slice, removed and either mixed with an equal volume of α-cyano-4-hydroxy-cinnamic acid solution (10 mg/ml in 30% acetonitrile, 0.1% trifluoroacetic acid) on a stainless steel MALDI target for initial MALDI MS measurement or diluted into 10 µl of 0.1% formic acid for subsequent LC-MS/MS measurements.

To obtain accurate *m/z* values for the *m/z* species of interest, matrix extracts from adjacent sections of mouse pancreata with a high intensity of the respective m/z species were analyzed by positive ion reflector mode MALDI MS. The measurements were performed on an ultrafleXtreme MALDI-TOF/TOF mass spectrometer equipped with a 1 kHz Smartbeam-II laser (Bruker Daltonics). Each spectrum was externally calibrated using the Peptide Calibration Standard II (Bruker Daltonics) and the “cubic enhanced” calibration function, typically yielding mass accuracy <20 ppm.

LC-MS/MS analysis of matrix extracts were performed on an LTQ Orbitrap mass spectrometer (Thermo Fisher Scientific) coupled to a nano-HPLC (nanoLC Ultra, Eksigent Technologies). Peptides were separated on a self-packed 75 µm ×40 cm reversed-phase column (Reprosil, Dr. Maisch) using a 25 min linear gradient (2–35% acetonitrile in 0.1% formic acid, flow rate 300 nl/min). Intact masses of eluting peptides were determined at 30,000 resolution and the three most intense peaks were selected for further fragmentation by collision-induced dissociation (CID) and acquisition of fragment spectra with low resolution (1,000). Singly charged ions as well as ions with unknown charge state were rejected. Dynamic exclusion was enabled and dynamic exclusion duration was set to 10 seconds. Peaklist files were generated using Mascot Distiller version 2.2.1.0 (Matrix Science) and database searches were performed using the Mascot search engine version 2.2.04 (Matrix Science) against the IPI mouse database (version 3.26). Search result files were imported into Scaffold (Proteome Software).

### Immunofluorescence and Immunohistochemistry

Immunofluorescence or immunohistochemistry were performed according to standard protocols. The following antibodies were used: ALB1 (Santa Cruz Biotechnology, and Thermo Fisher Scientific), HepPar1 (DAKO), TMSB4X [24], [25] (Immundiagnostics, Bensheim) and CK19 (TROMA-III; Developmental Studies Hybridoma Bank).

### Quantitative RT-PCR

Real-Time PCR was performed as previsouly described [8]. Cyclophilin was used for normalization. *P* values were calculated with the Wilcoxon test. The following primers were used:

Cyclophillin-F/−R ATGGTCAACCCCACCGTGT/TTCTGCTGTCTTTGGAACTTTGTC

Tmsb4x-F/−R CCTCTGCCTTCAAAAGAAACA/GGGCAGCACAGTCATTTAAAC

Alb1-F/−R TTGGTCTCATCTGTCCGTCA/GGCAGCACTCCTTGTTGACT

Transferrin-F/−R ATCAAGGCCATTTCTGCAAGT/GGTTCAGCTGGAAGTCTGTTCC

Alpha-Fetoprotein-F/−R GGAGGCTATGCATCACCAGT/CATGGTCTGTAGGGCTTTGC

Apolipoprotein A4-F/−R AGAGCCTGAGGGAGAAGGTC/AGGTGTCTGCTGCTGTGATG

### ELISA –Enzyme Linked Immunosorbent Assay

The ELISA kit for quantitative determination of TMSB4X concentrations in serum was obtained from Immundiagnostics (Bensheim). ELISA was performed according to the manufactureŕs protocol.

### Western Blot

Western Blot analysis was performed according to standard protocols with antibodies against ALB1 (Santa Cruz Biotechnology) and HSP90 (Cell Signaling).

## Results

### MALDI-IMS in Preneoplastic Lesions and PDAC of GEM Models

**Figure 1:**
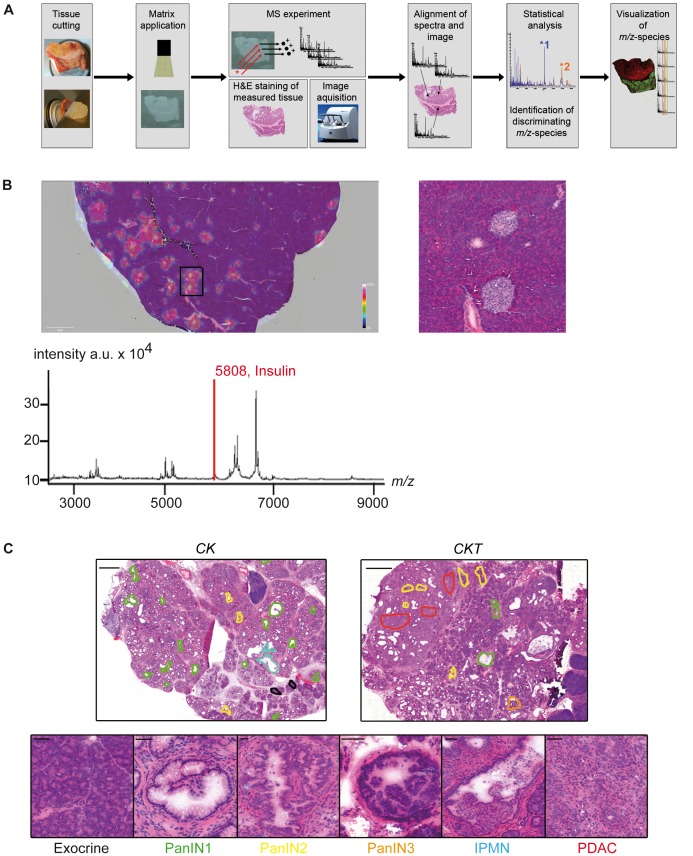
MALDI-IMS on wild type pancreas and definition of ROIs on H&E sections. (A) Overview of the MALDI-IMS workflow. (B) Re-visualization of the molecular ion of insulin [M+H^+^] at 5808 (red bar) in the average spectrum of a pancreatic section from a C57Bl/6 mouse measured in MALDI-IMS. The intensity of the measured signal is color coded, where red color means highest intensity at the regarding position on the section. The peak of insulin co-localizes with the pancreatic islets (magnified in excerpt). (C) Definition of ROIs on H&E stained sections after MALDI-IMS measurement. Upper panel: H&E stained sections of a *Ptf1a^+/Cre^;Kras^+/G12D^* (*CK*) and a *Ptf1a^+/Cre^;Kras^+/G12D^;Tgfa* (*CKT*) mouse. Black lines circle exocrine tissue, green lines PanIN1, yellow lines PanIN2, orange lines PanIN3, blue lines IPMN and red lines PDAC diagnosed regions in the respective section. Scale bar represents 1 cm. Lower panel: examples of the different morphological ROIs as indicated below. Scale bars represent 50 µm.

To identify novel biomarkers for the two most common preneoplastic pancreatic lesions, PanIN and IPMN, we resected pancreata from established GEM of PDAC: 13 *Ptf1a^+/Cre^;Kras^+/G12D^*, 8 *Ptf1a^+/Cre^;Kras^+/G12D^;Ela-Tgfα* and 5 *Ptf1a^+/Cre^;Kras^+/G12D^;Ela-Tgfa;Trp53^+/LSL-R172H^* mice of mixed age (3 to 18 month, depending on the genotype). These mice develop PanIN and IPMN lesions progressing to invasive and metastatic PDAC with different onset and aggressiveness [7], [8], [23]. Four C57Bl/6J mice served as wild type control. All pancreata were measured in an Ultraflex III MALDI TOF/TOF Analyzer with a spatial resolution of 70 µm. The MALDI-IMS workflow is depicted in Figure 1A. To test the accuracy of the method we first re-visualized the already known molecular ion of insulin [M+H^+^] at 5808 on pancreata of wild type mice. The insulin signal, which is given as a heat map illustration (where blue means lowest and red highest relative intensity), nicely co-localized with the islets of Langerhans (Figure 1B), demonstrating the correct correlation of measured *m/z*-species to morphological features with MALDI-IMS.

To compare the spectra of different morphological areas, regions of interest (ROI) for PanIN, IPMN, PDAC, and normal exocrine tissue were defined by an expert in pancreatic pathology on the pancreata using the FlexImaging software and were used for comparison of the spectra of respective regions from the same section as well as from other sections to each other. Figure 1C gives examples of the definition of regions on measured sections (Figure 1C upper panel) and the distinct morphological features (Figure 1C, lower panel). The single spectra of these ROIs were exported to ClinProTools analysis software. As a first control experiment we compared the spectra of normal pancreatic tissue (acini and ducts) from wild type (WT) mice with phenotypically normal appearing acinar and ductal tissue from *Ptf1a^+/Cre^;Kras^+/G12D^* mice harboring the oncogenic *Kras^G12D^* mutation. No differences in the spectra between these two groups were detectable, therefore ensuring that there are no detectable variances in the spectra of WT and GEM (Table 1). For further analysis, phenotypically normal ROIs from both genotypes were classified as “normal”.

**Table 1:** Statistical analysis of the different ROI groups for discriminating *m/z* species using ClinProTools.

compared groups	number of discriminating *m/z*-species	p values	Total number of animals (different genotypes)
Acini GEM vs Acini WT	0	–	6 vs 4
PanIN + IPMN vs normal	26 PanIN + IPMN	0.000001–0.05	24 vs 11
	50 normal		
PanIN vs normal	25 PanIN	0.00001–0.05	19 vs 11
	67 normal		
IPMN vs normal	18 IPMN	0.00005–0.05	13 vs 11
	28 normal		
PDAC vs normal	17 PDAC	0.0001–0.05	10 vs 11
	31 normal		
PanIN vs IPMN	6 PanIN	0.03–0.05	19 vs 13
	0 IPMN		
PanIN + IPMN vs PDAC	5 PanIN + IPMN	0.00169–0.037	24 vs 10
	11 PDAC		
IPMN vs PDAC	7 IPMN	0.01–0.05	13 vs 10
	2 PDAC		
PanIN vs PDAC	15 PanIN	0.00082–0.045	19 vs 10
	15 PDAC		

Listed are the compared groups, number of identified *m/z*-species specific for the indicated groups, the range of the corresponding *p-*values and the number of animals analyzed per group.

We next analyzed spectra from normal tissue of C57Bl/6J and *Ptf1a^+/Cre^;Kras^+/G12D^* mice (n = 11) against spectra from preneoplastic lesions of *Ptf1a^+/Cre^;Kras^+/G12D^* and *Ptf1a^+/Cre^;Kras^+/G12D^;Ela-Tgfα* mice (PanINs and IPMNs, n = 24). These two groups could be distinguished by 76 statistically significant peaks (Wilcoxon rank-sum test, *p* values Benjamini-Hochberg corrected) of which 26 were lesion-specific (i.e. specific for IPMNs and PanINs) and 50 normal-specific with *p* values between 0.000001 and 0.05. For PanINs we found 25 (*p* = 0.000001 to *p* = 0.05) and for IPMNs 18 (*p* = 0.03 to *p* = 0.05) specific *m/z*-species respectively, which could discriminate them from normal tissue. Also, IPMNs and PanINs could be discriminated from each other by 6 PanIN-specific peaks (*p* = 0.02 to *p* = 0.05, n = 19 vs. 13 mice). When comparing the preneoplastic lesions with PDAC (n = 24 vs. 10 mice) we detected 57 lesion-specific and 11 PDAC-specific masses (*p* = 0.00169 to *p* = 0.038). Table 1 provides an overview of all compared groups, the number of discriminating *m/z*-species, the corresponding *p* values and the number of animals used. Supplementary Table S1 provide detailed information of all significantly identified *m/z*-species of the most important comparisons.

### *m/z*-species 2790, 2812 and 2829 are Specifically Found in PanIN Lesions

**Figure 2:**
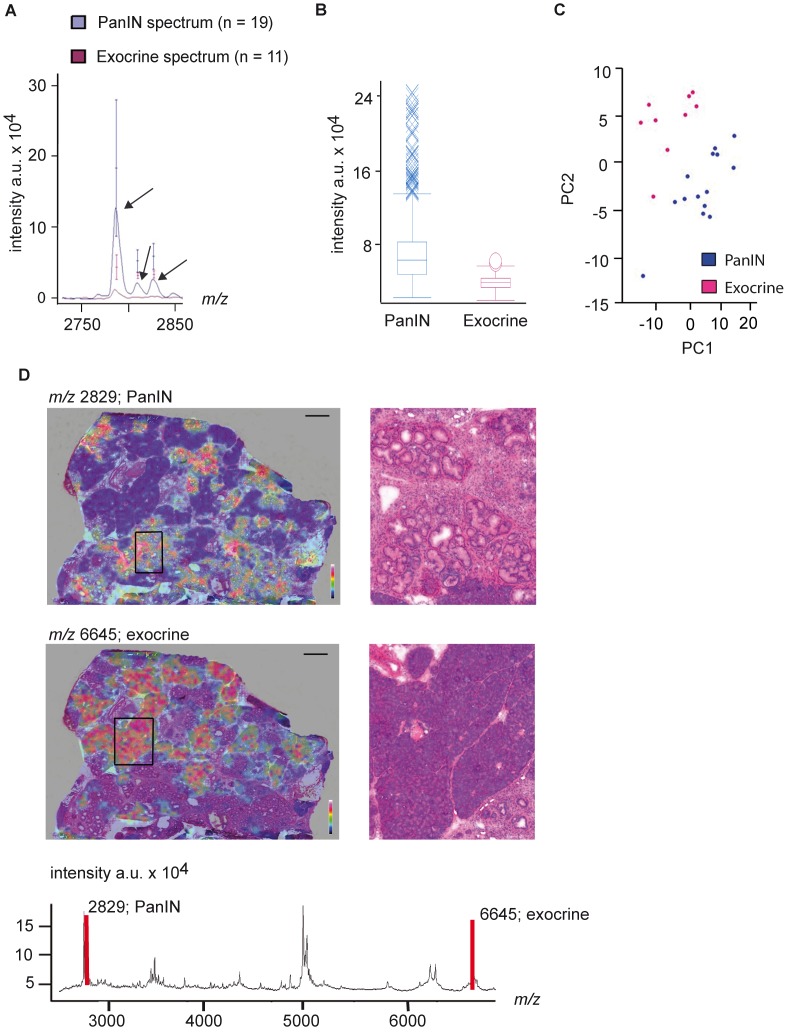
Statistical analysis of mass spectra comparison of “lesions” against “normal”. (A) Overlay of an excerpt of the average spectra from the ROI-group “PanIN” (blue) and the ROI-group “WT” (pink). The spread of the single spectra intensities is indicated in bars; a.u. (arbitrary units). (B) Dot Plot of the intensity-distribution of the *m/z*-species 2829 in PanINs (blue) and WT (pink) of each single spectrum. (C) PCA based differentiation of the exocrine (pink) and PanIN (blue) tissue. (D) Re-visualization of significant peaks. The *m/z*-species 2829 clearly re-visualizes on PanIN lesions (upper panel, magnified in excerpt) whereas the peak at 6645 is specific for the exocrine compartment of the pancreas (lower panel, magnified on the right side). Below is the average spectrum of the measured section.

Closer examination of PanIN-specific peaks revealed that the *m/z*-species 2790, 2812 and 2829 were discriminating PanINs from normal tissue (Figure 2A). The overlay of the average spectra from PanINs and normal pancreatic tissue revealed that in the latter the peaks were nearly not detectable (Figure 2A). Further statistical examination of these peaks (Wilcoxon test, Bonferroni correction) revealed *p* values below 0.00001. The distribution Box Plot and Principle Component Analysis (PCA) of PanINs and exocrine tissue depicted clear discrimination between the two groups (Figure 2B+C).

We next visualized *m/z* 2829 on the tissue sections demonstrating specificity of this peak for PanIN regions in the heat map illustration (Figure 2D, upper panel), whereas a peak at *m/z* 6645, which was unique for normal tissue specifically re-visualized in regions with morphologically normal pancreatic tissue (Figure 2D, lower panel).

### Validation of Significant Discriminating Peaks in an Independent Sample Set

To validate the significance of *m/z* species 2790 and 2829 in an independent sample set, we performed Receiver Operating Characteristics (ROC) analysis with these peaks to determine the optimal discriminating thresholds. With these thresholds it was possible to distinguish tissue from 10 independent mouse pancreata (4 *Ptf1a^+/Cre^;Kras^+/G12D^* and 6 wild type littermates at an age of 6 months) with an accuracy of 100% (Fisher test, *p*<0.001).

### Protein Identification of the Three Most Significant Species by LC-MS/MS

For protein identification of discriminating PanIN-specific significant *m/z* species, peptides were directly extracted from MALDI-IMS slides and initially analyzed by reflector mode MALDI MS to obtain accurate masses (<20 ppm) for the MALDI IMS species prior to LC-MS/MS analyses. Sequence database search of the LC-MS/MS results using the Mascot search engine allowed the identification of three highly significant *m/z* species pointing to two different proteins. The *m/z* 2790 species was identified as a peptide representing the amino-terminus of the mature form of serum Albumin (ALB1), whereas both, the *m/z* 2812 and the *m/z* 2829 species represented two different peptides belonging to the carboxy-terminus of Thymosin beta-4 (TMSB4X). The identification of TMSB4X was further supported by identification of an additional four different peptides of the protein’s carboxy-terminal region. The manually verified peptide identifications of ALB1 and TMSB4X and the corresponding MS/MS spectra are listed in Table 2 and available in the Supplemental Material (Figure S1).

**Table 2:** Overview of identified *m/z* species.

Significant MALDI-IMS species	Peptide sequence	Protein name	Mascot ionscore	Observed m/z	Calculated peptidemass/Da	Expected peptidemass/Da	Deviation/ppm
2790	EAHKSEIAHRYNDLGEQHFKGLVL	ALB1	65.2	931.1487	2790.4226	2790.4204	0.80
2812	SKLKKTETQEKNPLPSKETIEQEK	TMSB4X	32.5	704.1362	2812.5137	2812.5187	−1.77
2829	KTETQEKNPLPSKETIEQEKQAGES	TMSB4X	38.4	708.1087	2828.4037	2828.4044	−0.24
	KETIEQEKQAGES	TMSB4X	47.0	738.8627	1475.7098	1475.7106	−0.53
	KNPLPSKETIEQEKQAGES	TMSB4X	33.7	705.0310	2112.0696	2112.0702	−0.27
	KTETQEKNPLPSKETIEQEKQ	TMSB4X	36.3	829.0988	2484.2729	2484.2712	0.69
	KTETQEKNPLPSKETIEQEKQAG	TMSB4X	41.1	654.0902	2612.3296	2612.3298	−0.07

Depicted are the MALDI-IMS candidates, the identified peptide sequences and the proteins they belong to, as well as the corresponding Mascot ion scores of identification and the calculated and expected peptide masses with their deviation in ppm.

### Closer Investigation and Validation of Identified Candidates

**Figure 3:**
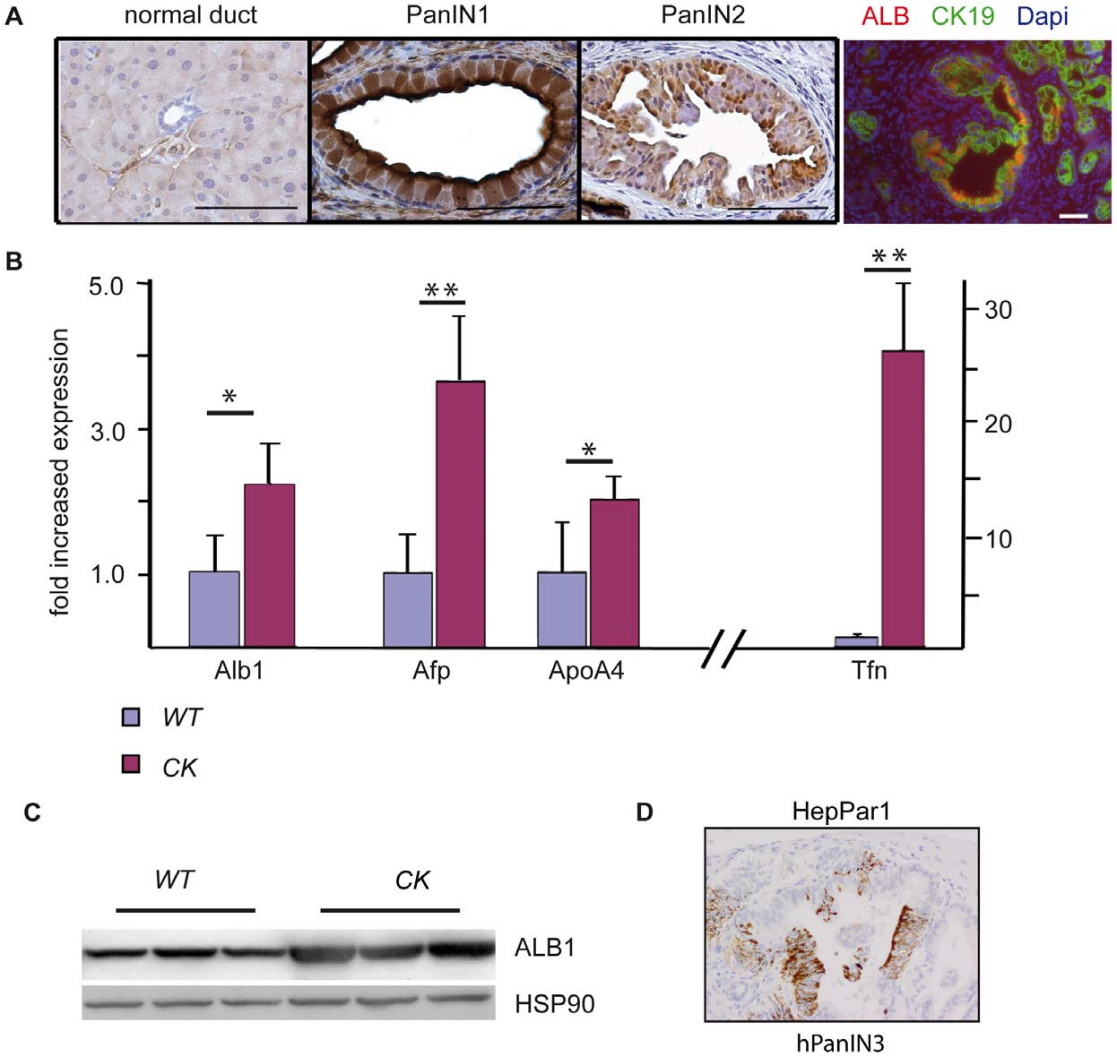
Identification and validation of ALB1. (A) Immunohistochemical analysis of ALB1 shows specific staining of PanIN lesions of *Ptf1a^+/Cre^;Kras^+/G12D^* (*CK*) mice but not ductal cells (n = 10 mice). Immunofluorescence staining for ALB1 and the ductal marker CK19 demonstrates co-localization of the two proteins. All scale bars represent 50 µm. (B) mRNA of *Alb1* and of the hepatic genes *Alpha-Fetoprotein (Afp)*, *Apolipoprotein A4 (ApoA4)* and *Transferrin (Tfn)* are all significantly upregulated in pancreata from *CK* mice compared to wild type control (*p* = 0.04 for *Alb1*, *p* = 0.008 for *Afp*, *p* = 0.04 for *ApoA4*, *p* = 0.004 for *Tfn*, n = 7 vs. 5 mice). Expression levels are normalized to samples of wild type mice. All error bars indicate the standard deviations normalized to the mean of the wild type. (C) Western Blot for ALB1 on whole pancreatic lysates from wild type and *CK* mice (n = 3). ALB1 expression in preneoplastic tissue is robustly increased comparing to normal pancreas. (D) Immunohistochemical analysis of the hepatic marker HepPar1 in a human PanIN3 lesion.

**Figure 4:**
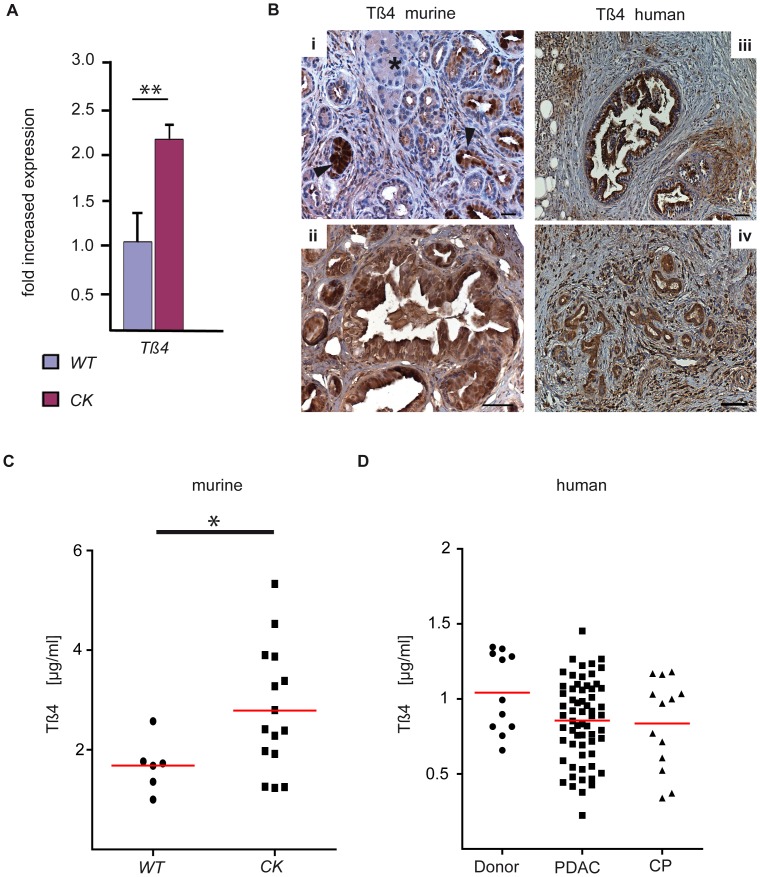
Expression analysis of TMSB4X. (A) TMSB4X mRNA is significantly increased in *Ptf1a^+/Cre^;Kras^+/G12D^ (CK)* mice compared to wild type control (*p* = 0.01, n = 8 vs. 6 mice). Expression levels are normalized to wild type. Error bars indicate the standard deviations normalized to the mean of the wild type. (B) Staining for TMSB4X on tissue samples from 10–30 week old *CK* mice (n = 10) shows expression in PanINs (arrowhead) but not in acinar (asterisk) cells (i). High-grade mPanIN3 express TMSB4X (ii). Expression in human PanIN3 (iii) and human PDAC (iv) is also detectable. Scale bars represent 50 µm. (C) ELISA for TMSB4X from serum samples of wild type and *CK* mice (n = 7 vs. 14 mice). The serum concentration of TMSB4X is significantly upregulated in *CK* mice (*p* = 0.043, Wilcoxon test). (D) ELISA for TMSB4X from serum samples of PDAC and CP patients as well as healthy donors. Medians are marked by red lines.

To investigate whether ALB1 and TMSB4X are also present on transcriptional level in tumorigenic pancreata, we isolated total pancreatic RNA from 8 *Ptf1a^+/Cre^;Kras^+/G12D^* and 6 wild type littermates at mixed age between 4.5 and 9 months and performed quantitative RT-PCR analysis for these two candidates. The expression of both transcripts was significantly upregulated in *Ptf1a^+/Cre^;Kras^+/G12D^* in comparison to wild type mice (*p*≤0.05, Figure 3B and 4A).

To validate correct ALB1 identification immunohistological staining and Western Blot analysis for ALB1 were performed. ALB1 expression on sections from *Ptf1a^+/Cre^;Kras^+/G12D^* mice was observed in PanIN lesions but not in normal pancreatic ducts and acinar cells (n = 10). Also, immunofluorescence analysis for ALB1 and the ductal marker CK19 on cryosections from *Ptf1a^+/Cre^;Kras^+/G12D^* and *Ptf1a^+/Cre^;Kras^+/G12D^;Ela-Tgfa* mice demonstrated co-localization of the two proteins in PanIN lesions (Figure 3A). Importantly, the *m/z* species 2790 did not re-visualize on small and large vessels of MALDI-IMS measured sections (Figure S2). Also Western Blot analysis of whole pancreatic lysates revealed increased ALB1 protein expression in *Ptf1a^+/Cre^;Kras^+/G12D^* and *Ptf1a^+/Cre^;Kras^+/G12D^* mice in comparison to wild type controls (Figure 3C).

It was previously reported that pancreatic exocrine cells can transdifferentiate to hepatocytes and that hepatic foci can be found in adult pancreas and in PDAC [26], [27], [28], [29]. Therefore we were intrigued to know whether the highly increased ALB1 signal identified by MALDI-IMS could be due to a hepatic transdifferentiation process of *Kras^G12D^*-activated pancreatic cells. To test this hypothesis we isolated RNA from whole pancreata of *Ptf1a^+/Cre^;Kras^+/G12D^* and wild type mice between 4.5 and 9 month and performed quantitative RT-PCR for the liver specific markers *Transferrin* (Tfn), *Alfa-fetoprotein* (Afp) and *Apolipoprotein A4* (ApoA4). The expression level of these markers were significantly increased in *Ptf1a^+/Cre^;Kras^+/G12D^* compared to wild type mice indicating a possible transdifferentiation process occurring in PanINs (*p*≤0.05, Figure 3B). Additionally we performed immunohistochemical analysis for the liver-specific marker HepPar1 on 14 PanIN1, 4 PanIN2 and 4 PanIN3 lesions. Two PanIN3 lesions were positively stained for HepPar1 (Figure 3D), indicating hepatic cell features in high-grade PanINs.

Regarding the second identified protein, we validated TMSB4X expression in murine and human PanIN lesions and PDAC but not in acinar, ductal and islet cells (Fig. 4B). For quantification we stained sections from 10 *Ptf1a^+/Cre^;Kras^+/G12D^* mice with PanINs and found all of them to be positive for TMSB4X. Of note, expression was high already in low-grade PanINs and stayed in malignant lesions, supporting the results of our MALID-IMS-based approach for identification of preneoplastic lesion markers that are still present in PDAC. Since TMSB4X is a small molecule and has been detected in body fluids previously, we next investigated whether it may be detectable by ELISA in serum samples of mice with PanIN lesions. Interestingly, we found significantly upregulated blood levels in *Ptf1a^+/Cre^;Kras^+/G12D^* compared to control WT mice, supporting the principal value of the presented marker detection strategy (Fig. 4C). However, when we performed an analysis using a set of human samples from donors and patients with chronic pancreatitis and PDAC, no significant difference was notable between these groups (Fig. 4D).

## Discussion

Because of the ongoing failure of therapeutic approaches to improve survival in PDAC patients, early detection is of key importance for better outcome in this otherwise fatal disease. In this study, we applied MALDI Imaging Mass Spectrometry (MALDI-IMS) with spatial resolution for *in situ* proteomic analysis of preneoplastic lesions of the pancreas in GEM with endogenous PDAC. We specifically addressed the question whether it is possible to identify proteins or peptides that can discriminate between morphologically normal pancreatic tissue, PanIN/IPMN precursor lesions and PDAC.

While the need for early detection of PDAC, ideally in a preinvasive state, is of obvious importance, proteomic analysis in humans are hindered by inherent interindividual and intratumoral genetic variations as well as confounding factors including environmental and nutritional conditions. In addition, obtaining pancreatic tissue with preneoplastic PanIN or IPMN lesions is not feasible for obvious reasons. Thus, GEM recapitulating human pancreatic carcinogenesis provide an excellent study platform and have been utilized for the detection of serum biomarkers using SELDI-TOF analysis [7]. In another study, *Pdx1-Cre;Kras^+/G12D^;Ink4a/Arf^lox/lox^* mice were used for plasma proteomic analysis and candidates were validated in the blood of patients with PDAC [30]. A recent study from Taguchi and colleagues compared plasma protein profiles of four mouse models of lung cancer with profiles of models of pancreatic, ovarian, colon, prostate, and breast cancer and two models of inflammation. They showed relevance to human lung cancer of the protein signatures identified on the basis of mouse models [31]. We therefore hypothesized these GEM to be a suitable platform for biomarker identification using MALDI-IMS.

MALDI-IMS is a rapidly developing approach for molecular tissue analysis with high potential for clinically relevant questions including identification of biomarkers, tumor classification, therapy response monitoring and drug imaging [14], [16], [32], [33], [34], [35], [36]. In comparison to conventional mass spectrometry, a major advantage of this technique is the possibility of histology-directed tissue profiling with localization of identified *m/z*-species to specific tissue compartments such as preneoplastic lesions.

Laser-capture microdissection (LCM) followed by shotgun proteomics is a powerful alternative for the analysis of tissue sections, and routinely enables the identification of hundreds of proteins from low numbers of cells. However, in contrast to MALDI IMS, LCM-based proteomics does not retain the information of the spatial distribution of different analytes on the tissue. Moreover, LCM targets specific tissue compartments, while MALDI IMS enables a global view of all different tissue types and morphologies present on the measured section.

The spatial resolution of 70 µm as used in this study is clearly far away from the resolution achieved in conventional histological and light-microscopical analyses or with LCM. However, it still allowed the analysis of small PanIN lesions and even larger normal ducts, enabling us to identify m/z species selectively expressed in the respective compartments. At present, spatial resolution in MALDI IMS of 25 µm can be achieved and is mostly limited by matrix crystal size [37]. Further, the employed resolution is a compromise of laser beam size and ion yield, with narrower laser beams enabling higher resolution but lower peak intensities.

At the current stage, MALDI IMS usually covers a range of detection from 500 to 25,000 *m/z* [38], thereby inevitably excluding a large proportion of the intact proteome. Nonetheless, about 150 *m/z* species could be observed and spatially resolved in the present study. Among the differentially expressed *m/z* species, we found *m/z* 2790, 2812 and 2829 to be highly specific for PanIN lesions. This enrichment was validated in an independent test sample of pancreatic tissue with PanIN-bearing vs. normal pancreatic tissue, demonstrating the discriminatory ability of the identified peaks.

Clearly, the identification of proteins behind the discriminant peaks observed in MALDI IMS still represents a major bottleneck of this technique and no routine method for this task is available. Moreover, most MALDI IMS studies are confined to elaborate statistical analyses and only in rare cases report protein identifications. Here, we identified two proteins, namely ALB1 and TMSB4X, which represent statistically significant MALDI IMS peaks. Given the small amount of sample (i. e. 1 µl of matrix extract) used for the LC-MS/MS analysis, it is not surprising to identify solely peptides originating from abundant proteins. Future methodological refinements and more sensitive MS instruments may enable the routine identification of many more interesting MALDI IMS species.

TMSB4X is a protein known to be upregulated in human PDAC cells [39], [40] and in the developing pancreas [41]. Recent functional and expression studies suggest an important role of this protein during organogenesis and in many cellular processes including progenitor cell regulation [25], [42]. While functional analysis is beyond the scope of this study, TMSB4X may play a role in early preneoplastic and/or progenitor cell transformation under oncogenic stress. Interestingly, it has been identified in proteomic screens in various diseases and tissues, probably because of its small size, cleavage and high expression levels.

In addition to TMSB4X, ALB1 was identified from the mass 2790. Both candidates were analysed with quantitative RT-PCR in total pancreatic lysates, demonstrating a significant upregulation also of the transcripts of these proteins in *Ptf1a^+/Cre^;Kras^+/G12D^* mice in a preneoplastic state. The confirmation of increased ALB1 and TMSB4X expression on protein level using Western Blot and immunohistological stainings in murine and human PanIN lesions verifies the principal ability of MALDI-IMS and subsequent LC-MS/MS analysis to identify the respective proteins or peptides from peaks measured *in situ*. These results demonstrate the utility of the method to identify potential biomarkers even from small amounts of tissue such as PanIN lesions. ALB1 has previously been identified to be present in pancreatic tumor tissue sections by direct MALDI-IMS-MS/MS [43]. Its identification in PanIN lesions may be due to several reasons. Potentially, ALB1 may be attracted and bound by the mucinous content of PanIN lesions as has been described for the transepithelial transport of serum proteins to the intestinal mucus [44]. While identification of murine ALB1 rules out contamination by fetal calf serum, serum ALB1 from blood vessels may potentially be recruited or associate with PanINs. However, in our view this is an unlikely scenario especially since *m/z* 2790 was not detectable in blood vessels. Alternative possibilities include expression of ALB1 from quiescent pancreatic stellate cells [45], located in the stroma surrounding the PanIN lesions. However, we did not detect ALB1 expression by re-visualization or by immunohistochemistry in the PanIN-surrounding stromal tissue. Detecting ALB1 specifically in PanIN lesions may therefore suggest a regulatory mechanism that warrants further investigation. Previous reports have shown that pancreatic exocrine cells can transdifferentiate to hepatocytes and that hepatic foci can be found in adult pancreas and in PDAC [26], [27], [28], [29]. The transdifferentiated hepatocyte-like cells express a variety of proteins normally present in mature hepatocytes among which are ALB1, acute phase proteins and the liver-specific markers Transferrin, Alpha-Fetoprotein and Apolipoprotein A4 [46], [47], [48]. In a recent paper, MacDonald and colleagues provide evidence that acinar cells start to express liver-restricted genes after modulation of the acinar cell specifying complex PTF1 [49]. Thus, activation of oncogenic Kras^G12D^ in acinar cells may lead to downregulation of PTF1 activity and subsequent expression of liver-restricted genes.

The confirmation of increased TMSB4X expression in sera from mice harboring preneoplastic lesions verifies the principal ability of MALDI-IMS and subsequent LC-MS/MS analysis to identify the respective proteins or peptides from peaks measured *in situ*. However, the subsequent approach of its utility as human serum biomarker for PDAC identification failed to show significant differences, although staining for TMSB4X on human tissue depicted specific expression in PanINs and PDAC tissue. Thus, it likely represents an imperfect biomarker. Nevertheless, this approach demonstrates the principal utility of the method to identify potential biomarkers in this disease and future work will focus on identifying additional proteins from the identified masses.

Obviously, clinically useful biomarkers need to be measured distantly, i.e. in easily accessible body fluids like serum or pancreatic juice, to ensure applicability of screening approaches. ALB1 is no suitable candidate for obvious reasons, while TMSB4X is a protein that has been identified in a variety of pathological conditions, potentially arguing against this protein as specific enough within the aimed clinical context. The identification of many lesion-specific peaks of yet unknown proteins may hopefully lead to the detection of clinically meaningful biomarkers. In addition, with the advantage of a spatially-resolved proteomic approach, this method is suitable to define lesion-specific protein signatures, which is subject of future studies. Our approach as a proof-of-concept study may be valuable for several reasons: (i) it is one of the very few studies that have identified proteins from masses; (ii) we have identified several and in some instances a long list of significant discriminating peaks when comparing the various lesion subtypes and disease conditions, enabling the future identification of potentially more suitable biomarker candidates; (iii) although PanIN- or IPMN-specific proteins may not be detectable in peripheral blood, they may still be detectable in other compartments such as pancreatic juice or cyst fluid helping to identify patients at risk for harboring preneoplastic pancreatic lesions. As such we consider MALDI-IMS on sections from endogenous mouse models of PDAC a valuable approach for proteomic investigations of diseased tissue.

## Supporting Information

Figure S1 **Supplemental information on peptide and protein identifications by LC-MS/MS.**
(DOC)

Figure S2 **Re-visualization of the** ***m/z*** **species 2790.**
(TIF)

Table S1 **Listing of all significant** ***m/z*** **-species from the various comparisons.**
(DOC)
