# Supplementary material for: MALDI Imaging Mass Spectrometry for In Situ Proteomic Analysis of Preneoplastic Lesions in Pancreatic Cancer
Source: PLoS One. 2012 Jun 26;7(6):e39424. doi: 10.1371/journal.pone.0039424 (PMC3383687; doi:10.1371/journal.pone.0039424)
Supplement: Figure S1 — Supplemental information on peptide and protein identifications by LC-MS/MS. (DOC) [file pone.0039424.s001.doc]

**Supplemental Figure S1**

**Supplemental information on peptide and protein identifications by LC-MS/MS**

The murine proteins ALB1 (serum Albumin) and TMSB4X (Thymosin 4-beta) were identified by direct peptide extraction from MALDI-IMS slides and subsequent LC-MS/MS analysis and sequence database search using the Mascot search engine.

**Annotated MS/MS spectra supporting the identification of ALB1 and TMSB4X**

1. ALB1 EAHKSEIAHRYNDLGEQHFKGLVL (parent *m/z* 931.1487, Mascot ion score 65.2)

**
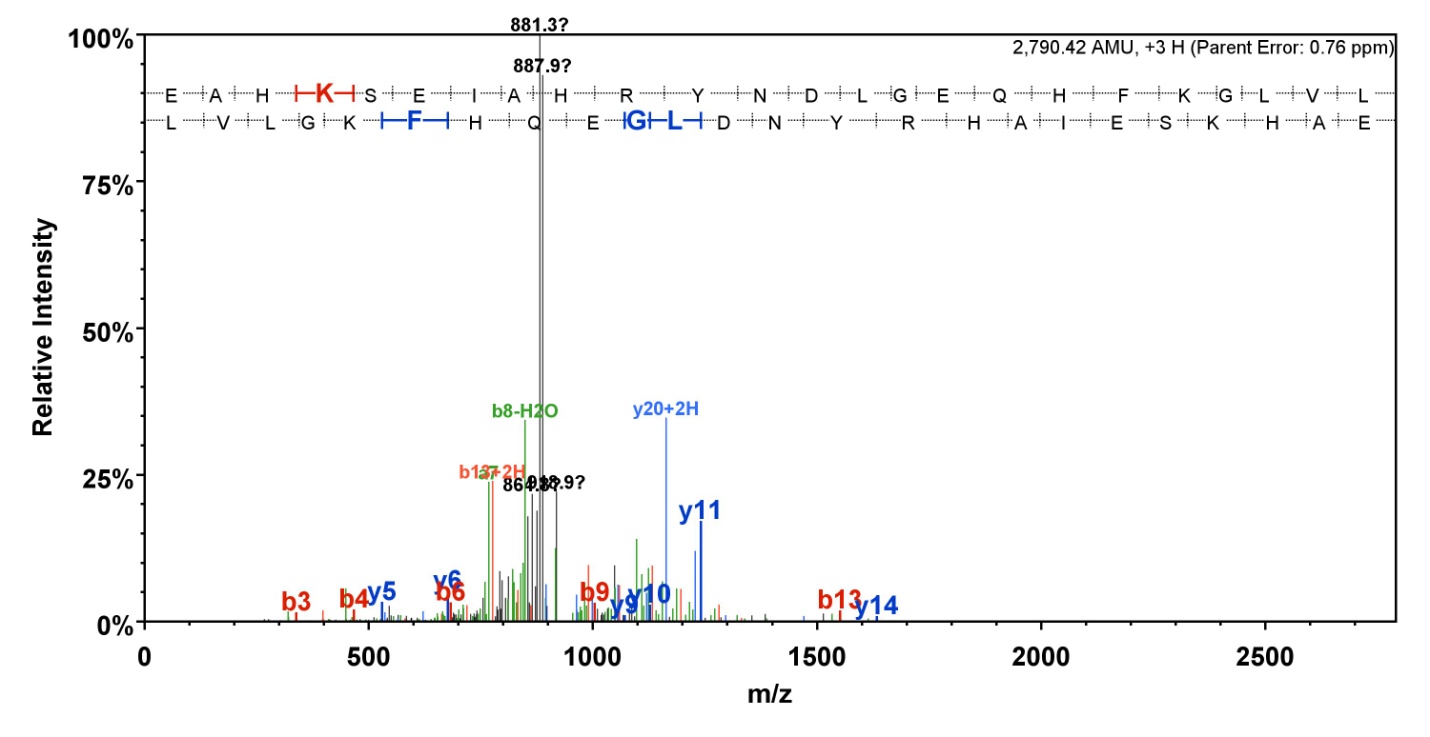
**

2. TMSB4X SKLKKTETQEKNPLPSKETIEQEK (parent *m/z* 704.1362, Mascot ion score 32.5)


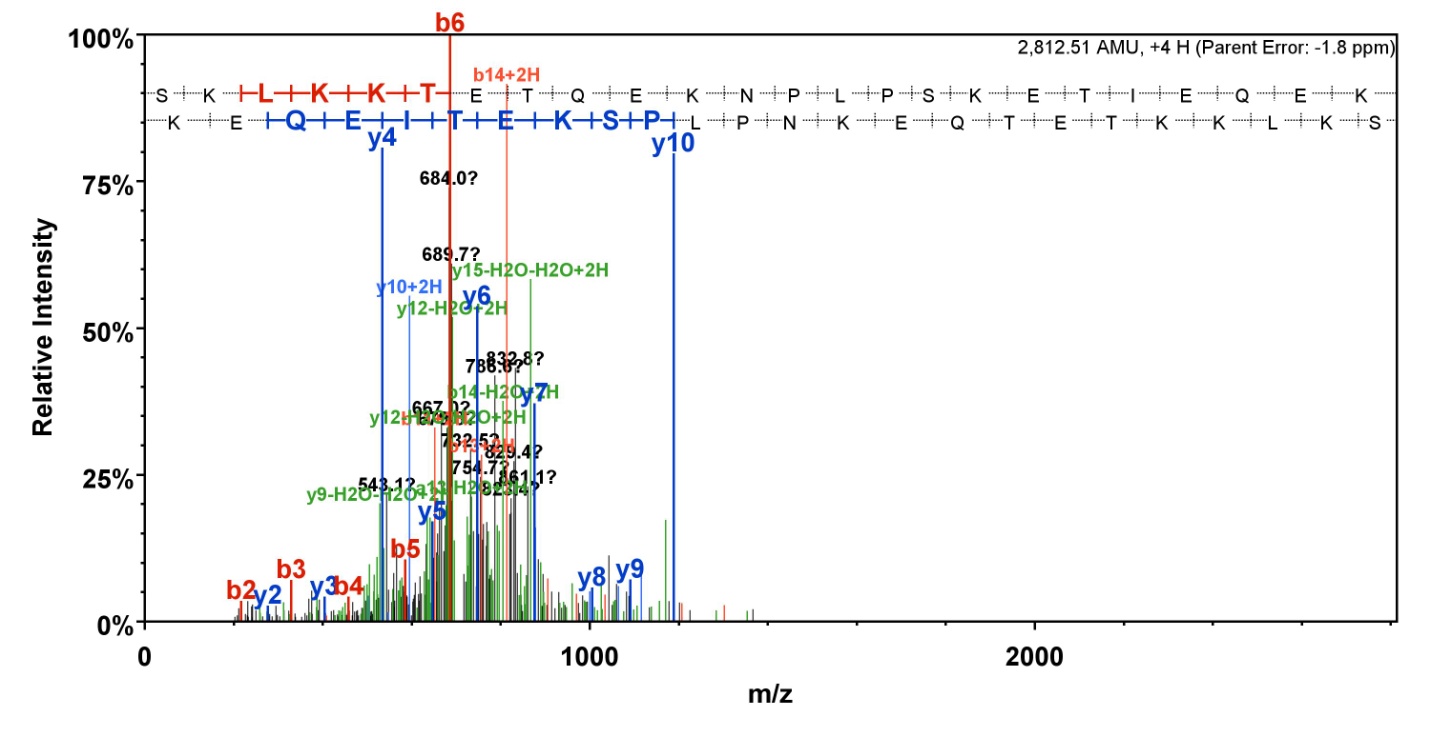


1. TMSB4X KTETQEKNPLPSKETIEQEKQAGES (parent *m/z* 708.1087, Mascot ion score 38.4)


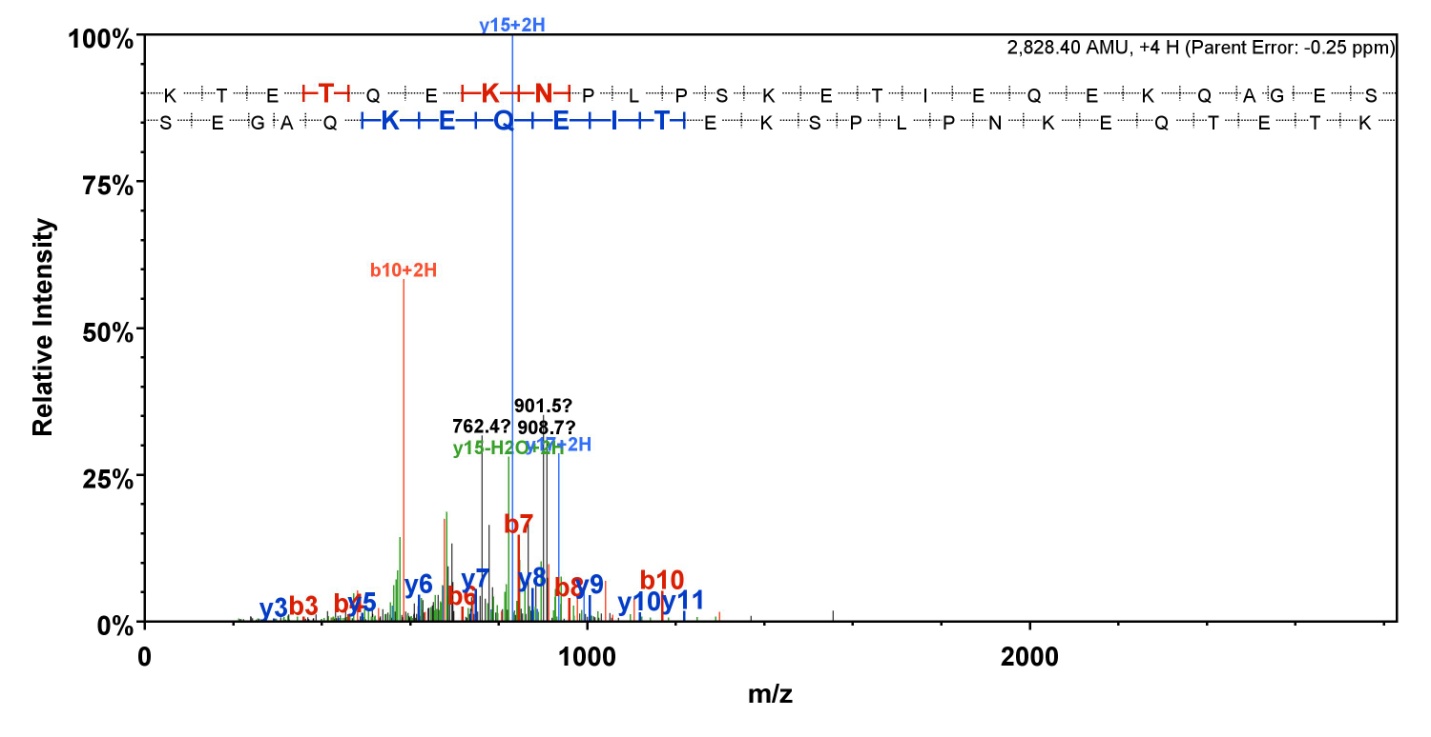


1. TMSB4X KETIEQEKQAGES (parent *m/z* 738.8627, Mascot ion score 47.0)


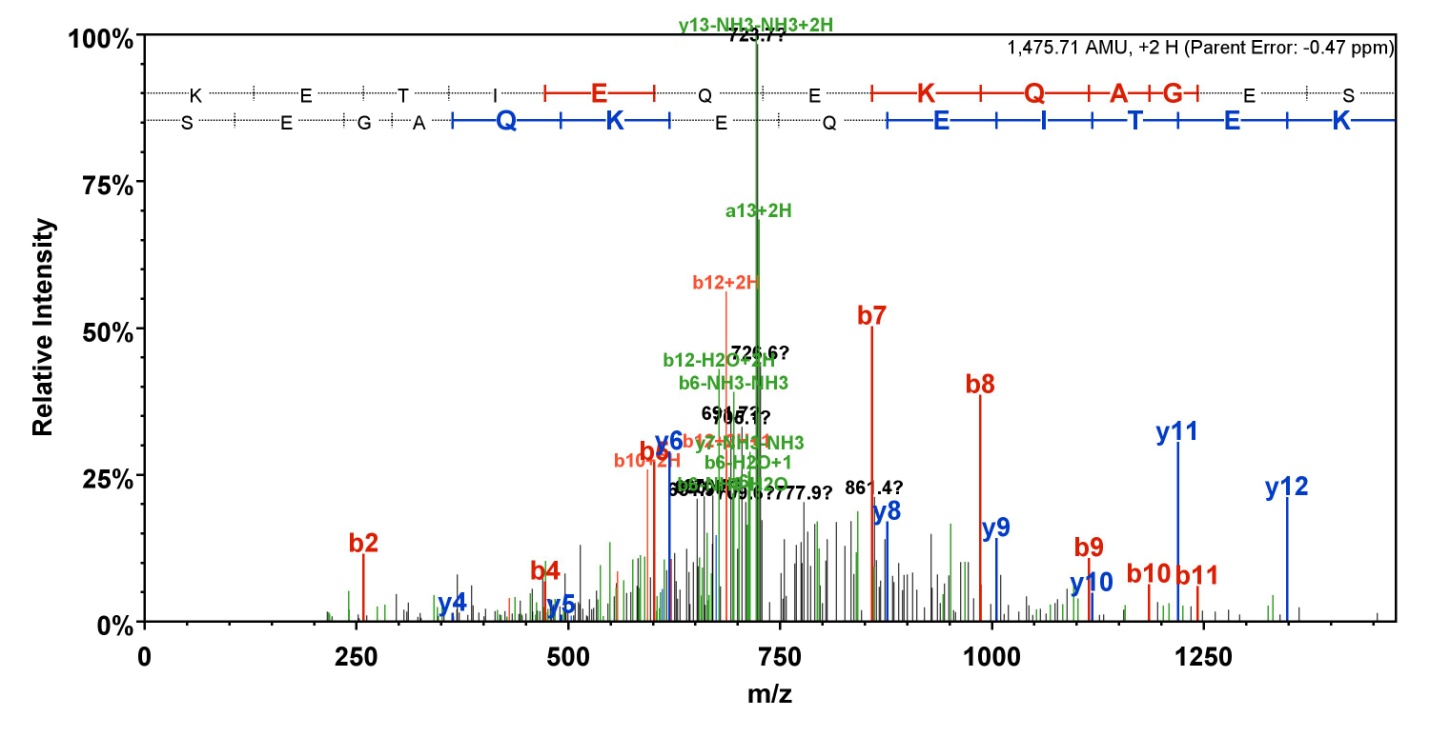


1. TMSB4X KNPLPSKETIEQEKQAGES (parent m/z 705.0310, Mascot ion score 33.7)


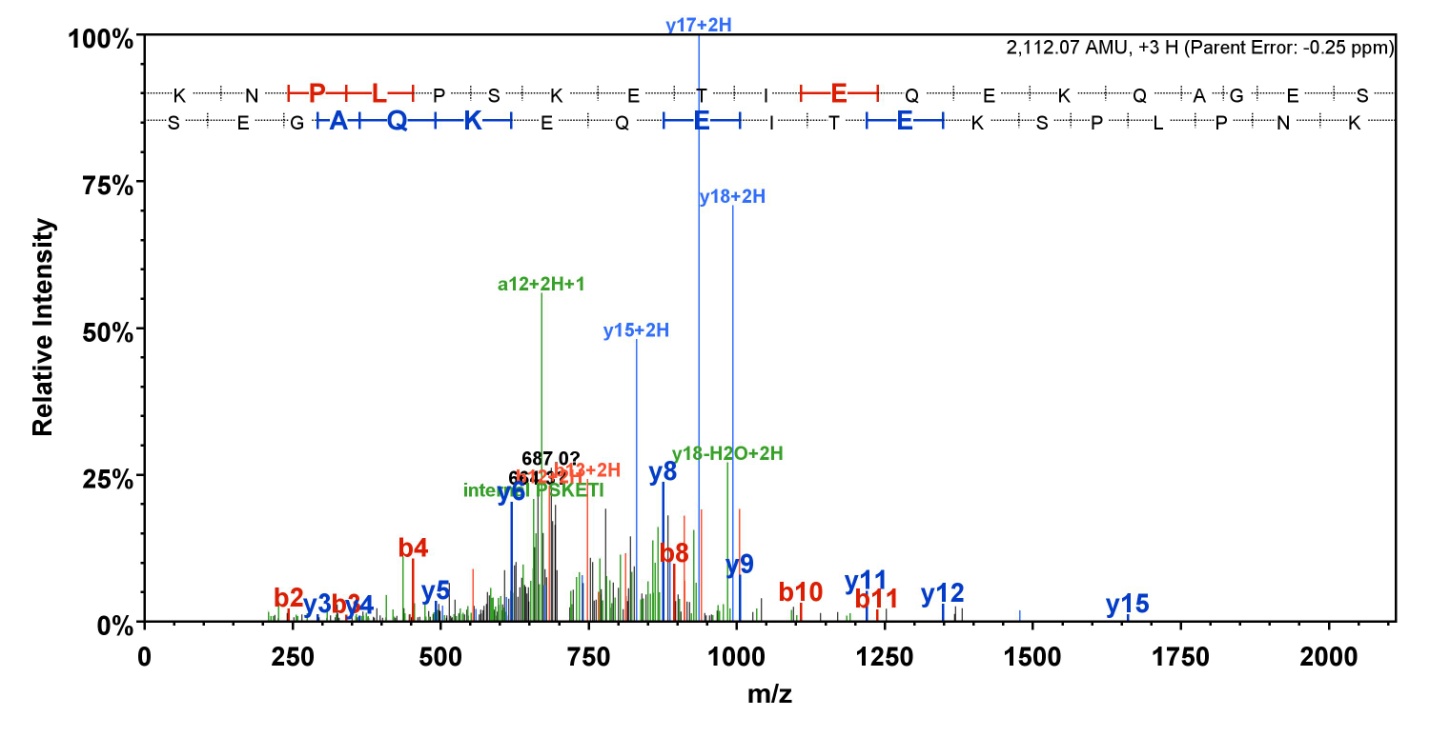


1. TMSB4X KTETQEKNPLPSKETIEQEKQ (parent *m/z* 829.0988, Mascot ion score 36.3)


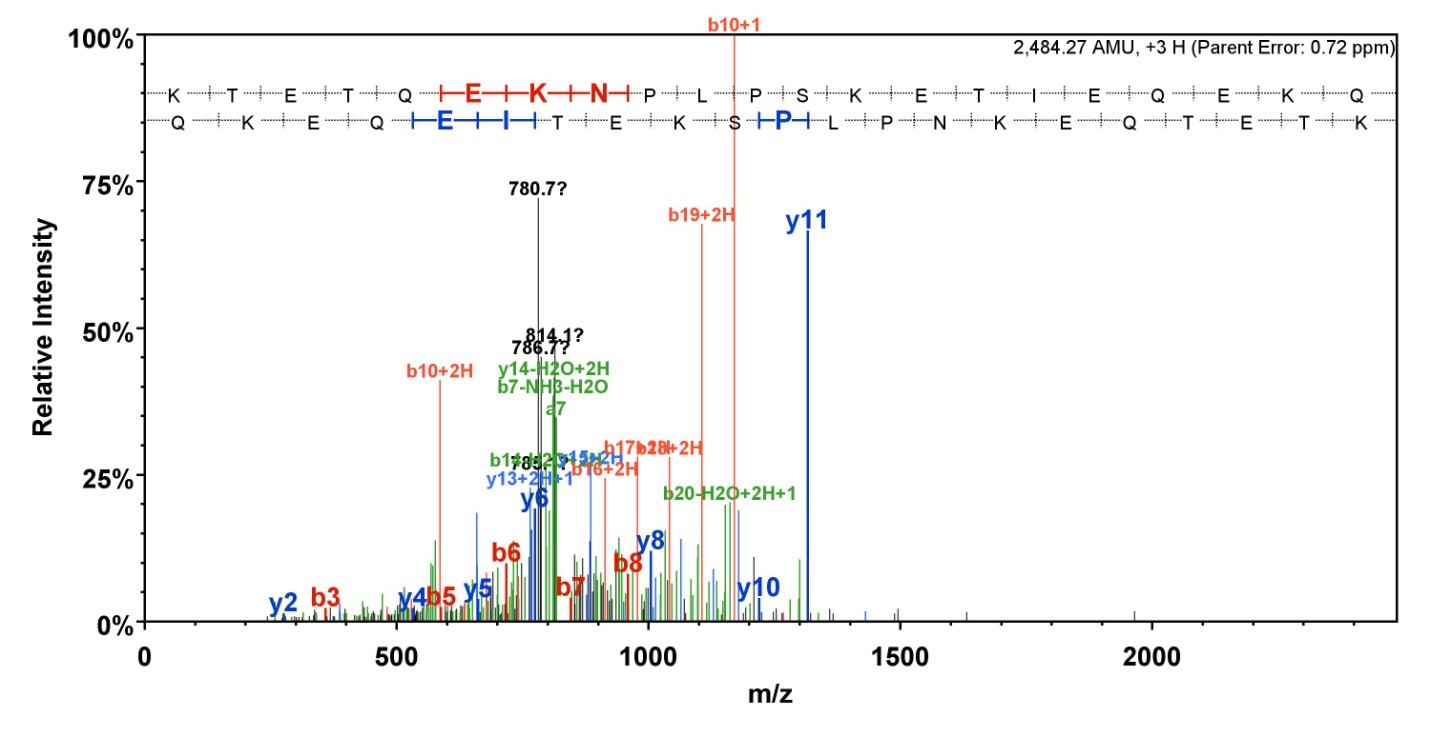


1. TMSB4X KTETQEKNPLPSKETIEQEKQAG (parent m/z 654.0902, Mascot ion score 41.1)

**
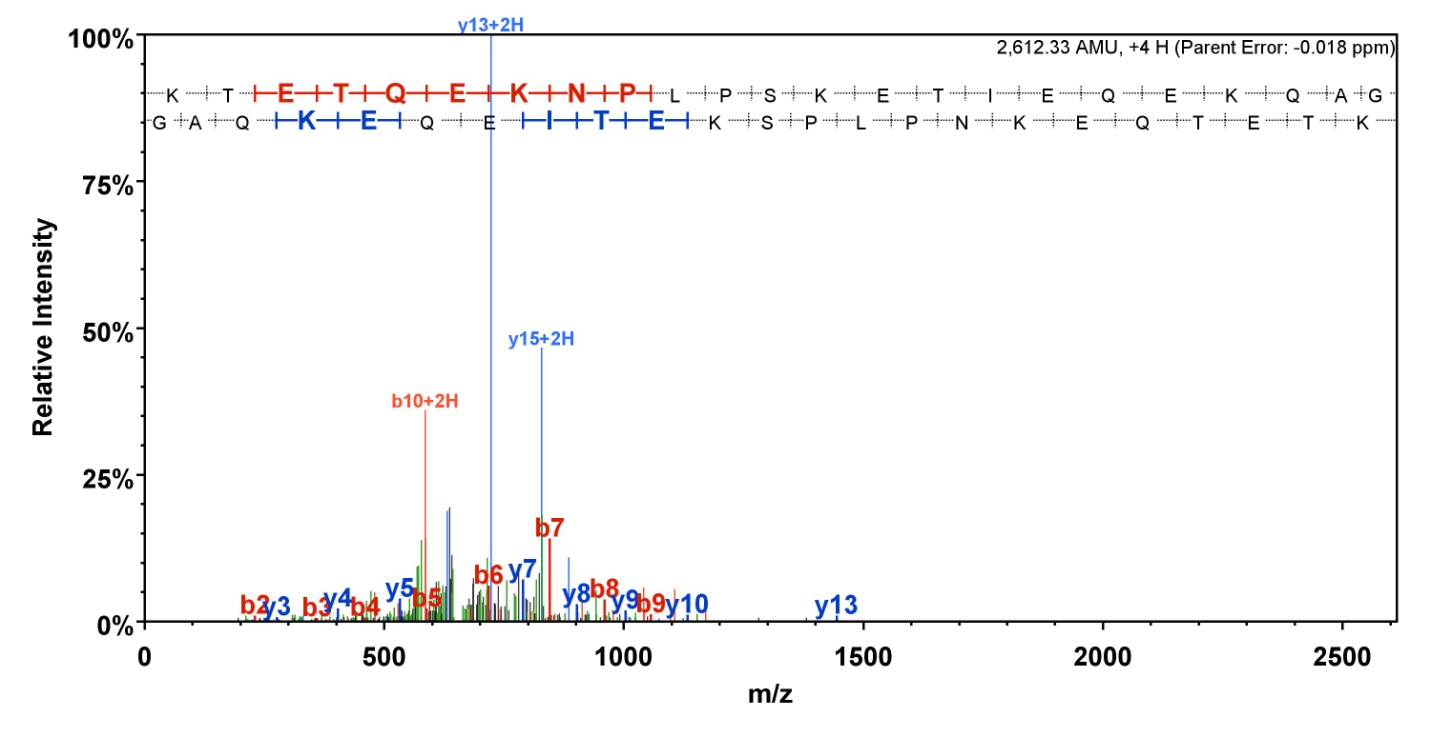
**

**Sequence coverage of ALB1 and TMSB4X**

**
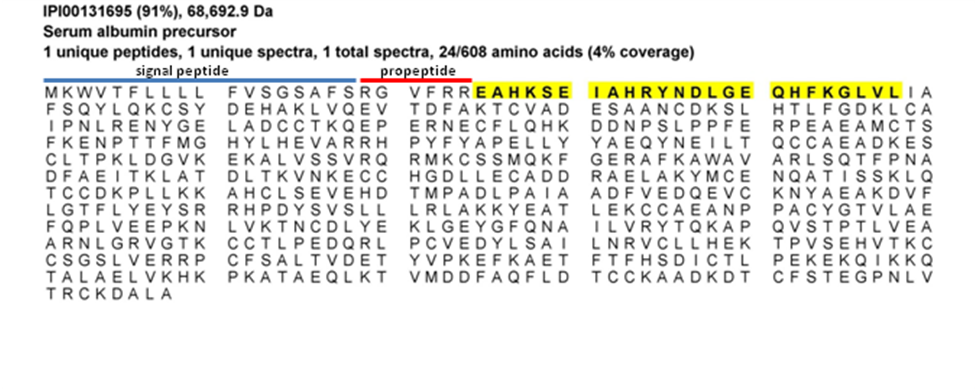
**

**
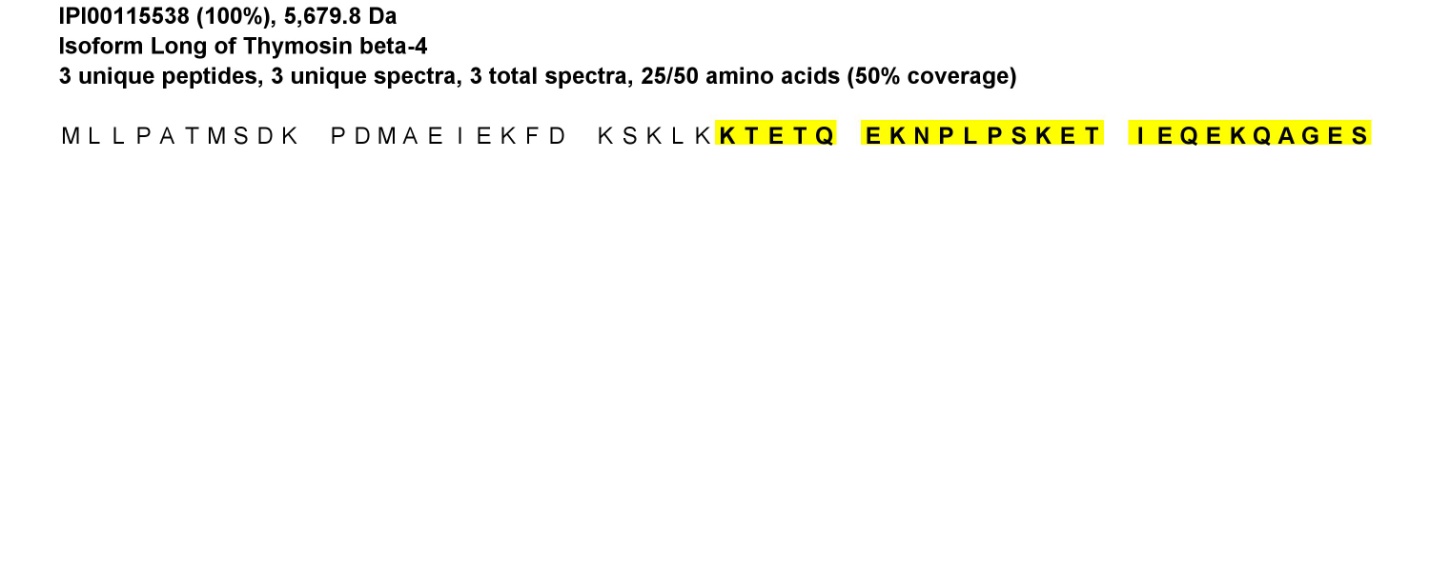
**
