# Supplementary figures and images for: MALDI Imaging Mass Spectrometry for In Situ Proteomic Analysis of Preneoplastic Lesions in Pancreatic Cancer
Source: PLoS One. 2012 Jun 26;7(6):e39424. doi: 10.1371/journal.pone.0039424 (PMC3383687; doi:10.1371/journal.pone.0039424)

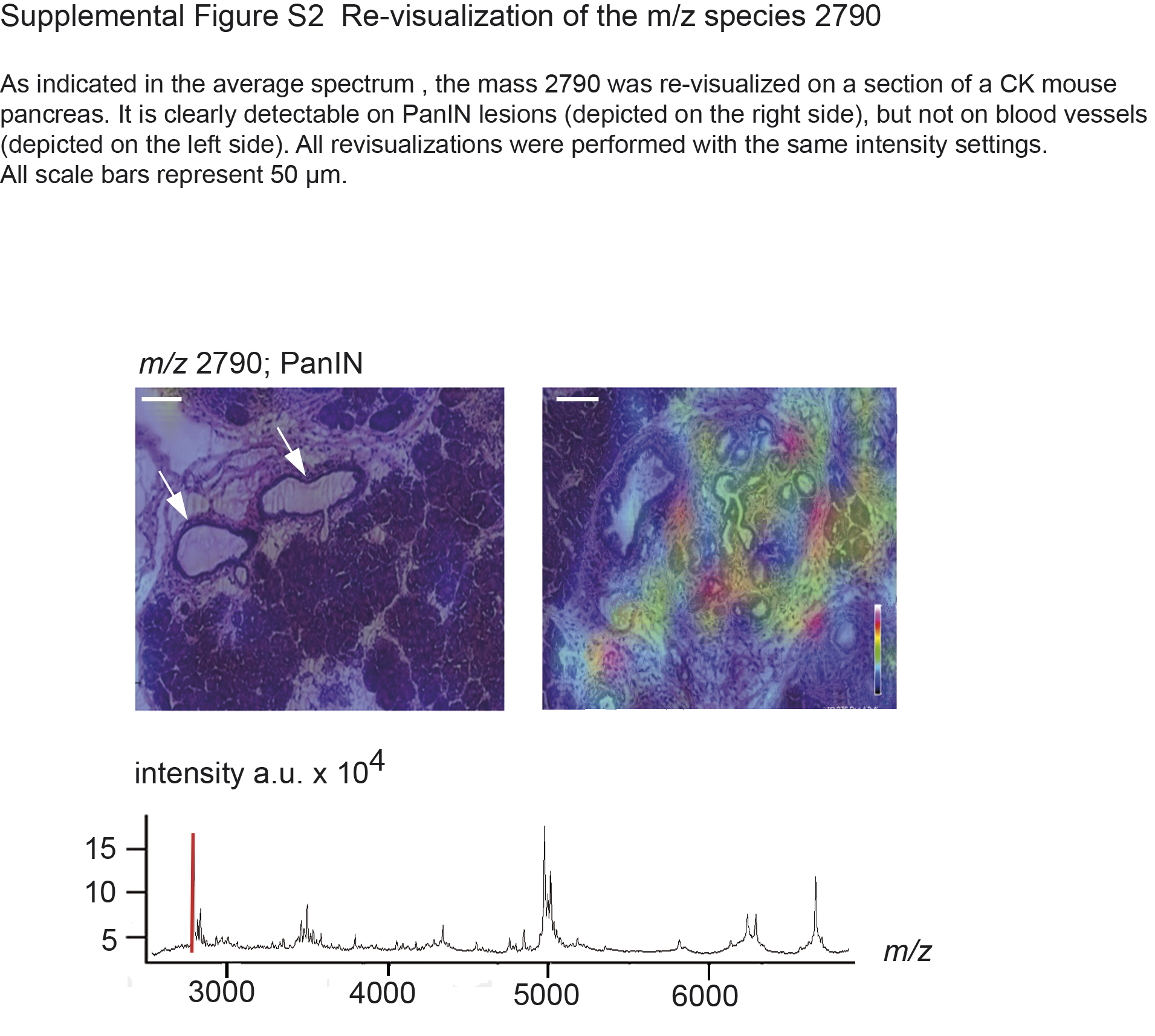

Supplement: Figure S2 — Re-visualization of the m/z species 2790. (TIF) [file pone.0039424.s002.tif]
