# Supplementary material for: MALDI Imaging Mass Spectrometry for In Situ Proteomic Analysis of Preneoplastic Lesions in Pancreatic Cancer
Source: PLoS One. 2012 Jun 26;7(6):e39424. doi: 10.1371/journal.pone.0039424 (PMC3383687; doi:10.1371/journal.pone.0039424)
Supplement: Table S1 — Listing of all significant m/z -species from the various comparisons. (DOC) [file pone.0039424.s003.doc]

**Tables S1 Listing of all significant *m/z*-species from the various comparisons**

Each table lists all discriminating *m/z*-species (mass) for the in the table title indicated comparison, the corresponding *P* value with Wilcoxon rank-sum test with Benjamini-Hochberg correction (PWKW) and the average relative intensity (AVE) of this mass within the two groups. For reasons of clarity and comprehensibility *P* values were rounded according to standard mathematical rules.

**Significant *m/z*-species from comparison PanIN vs IPMN**

| **Mass** | **PWKW** | **Ave IPMN** | **Ave PanIN** |
| --- | --- | --- | --- |
| 2813.18 | <0.05 | 5.33 | 7.83 |
| 2830.11 | <0.05 | 5.49 | 7.71 |
| 3789.68 | <0.05 | 3.79 | 5.68 |
| 7219.74 | <0.05 | 2.1 | 3.69 |
| 7418.39 | <0.05 | 2.2 | 3.72 |
| 2791.08 | <0.05 | 18.1 | 27.56 |

**Significant *m/z*-species from comparison PanIN vs PDAC**

| **Mass** | **PWKW** | **Ave PanIN** | **Ave PDAC** |
| --- | --- | --- | --- |
| 4936.62 | <0.001 | 4.28 | 12.28 |
| 3473.41 | <0.001 | 6.63 | 3.88 |
| 3438.84 | <0.001 | 5.21 | 3.46 |
| 3789.66 | <0.01 | 5.66 | 3.42 |
| 7219.74 | <0.01 | 3.71 | 1.9 |
| 7180.42 | <0.01 | 2.14 | 1.64 |
| 7418.46 | <0.01 | 3.75 | 1.89 |
| 6223.02 | <0.01 | 3.35 | 4.61 |
| 5836.12 | <0.05 | 3.68 | 2.29 |
| 3531.57 | <0.05 | 4.99 | 3.86 |
| 4747.02 | <0.05 | 3.66 | 6.67 |
| 4334.14 | <0.05 | 7.35 | 5.16 |
| 9959.11 | <0.05 | 1.44 | 2.82 |
| 6274.23 | <0.05 | 3.53 | 4.89 |
| 3514.79 | <0.05 | 4.57 | 3.74 |
| 4165.71 | <0.05 | 4.33 | 3.51 |
| 14034.25 | <0.05 | 0.92 | 1.73 |
| 11300.51 | <0.05 | 0.99 | 1.47 |
| 6257.68 | <0.05 | 2.39 | 2.89 |
| 4319.12 | <0.05 | 4.05 | 3.24 |
| 4236.97 | <0.05 | 4.29 | 3.63 |
| 8425.64 | <0.05 | 1.54 | 1.83 |
| 4963.06 | <0.05 | 17.2 | 37.17 |
| 5800.84 | <0.05 | 6.24 | 2.54 |
| 11337.21 | <0.05 | 0.98 | 1.31 |
| 3455.95 | <0.05 | 5.03 | 4.03 |
| 14001.79 | <0.05 | 0.96 | 1.57 |
| 6242.88 | <0.05 | 2.63 | 3.16 |
| 6170.3 | <0.05 | 2.4 | 2.69 |
| 4984.01 | <0.05 | 7.81 | 13.05 |

**Significant *m/z*-species from comparison PDAC vs IPMN**

| **Mass** | **PWKW** | **Ave PDAC** | **Ave IPMN** |
| --- | --- | --- | --- |
| 4936.53 | < 0.01 | 12.3 | 4.51 |
| 3456.02 | < 0.01 | 4.03 | 5.89 |
| 3423.61 | < 0.01 | 3.7 | 4.5 |
| 3514.57 | < 0.01 | 3.74 | 4.74 |
| 3530.81 | < 0.05 | 3.83 | 5.04 |
| 3473.17 | < 0.05 | 3.84 | 5.37 |
| 3439.16 | < 0.05 | 3.49 | 4.97 |
| 9958.4 | < 0.05 | 2.83 | 1.39 |
| 5765.04 | < 0.05 | 2.07 | 2.93 |

**Significant *m/z*-species from comparison PDAC vs Lesion (PanIN and IPMN)**

| **Mass** | **PWKW** | **Ave Lesion** | **Ave PDAC** |
| --- | --- | --- | --- |
| 4936.44 | < 0.001 | 4.31 | 12.28 |
| 3424.45 | < 0.01 | 4.58 | 3.72 |
| 3473.09 | < 0.01 | 5.64 | 3.87 |
| 3439.43 | < 0.01 | 4.82 | 3.48 |
| 3530.74 | < 0.01 | 5.04 | 3.84 |
| 7417.9 | < 0.05 | 3.12 | 1.89 |
| 3789.51 | < 0.05 | 5.01 | 3.42 |
| 5835.38 | < 0.05 | 3.49 | 2.22 |
| 9958.48 | < 0.05 | 1.49 | 2.85 |
| 7219.16 | < 0.05 | 3.1 | 1.91 |
| 3455.73 | < 0.05 | 5.19 | 4.03 |
| 3514.25 | < 0.05 | 4.57 | 3.74 |
| 4962.82 | < 0.05 | 17.85 | 37.17 |
| 4746.61 | < 0.05 | 4.02 | 6.68 |
| 14033.43 | < 0.05 | 1.01 | 1.73 |
| 5800.94 | < 0.05 | 5.66 | 2.44 |

**Significant *m/z*-species from comparison wild type vs PDAC**

| **Mass** | **PWKW** | **Ave WT** | **Ave PDAC** |
| --- | --- | --- | --- |
| 6646.03 | < 0.001 | 19.9 | 4.77 |
| 6683.87 | < 0.001 | 5.33 | 2.13 |
| 6604.23 | < 0.001 | 3.68 | 2.13 |
| 6310.97 | < 0.001 | 3.75 | 2.41 |
| 6571.18 | < 0.001 | 2.55 | 2 |
| 3454.92 | < 0.001 | 7.91 | 4.11 |
| 6118.98 | < 0.001 | 3.62 | 2.35 |
| 2791.7 | < 0.001 | 5.83 | 29.41 |
| 4936.6 | < 0.001 | 3.48 | 12.29 |
| 6274.16 | < 0.001 | 10.71 | 4.9 |
| 6222.46 | < 0.001 | 9.07 | 4.62 |
| 6142.17 | < 0.001 | 2.86 | 2.19 |
| 6189.29 | < 0.001 | 3.98 | 2.61 |
| 3002.06 | < 0.001 | 3.76 | 5.01 |
| 6851.29 | < 0.001 | 2.32 | 1.8 |
| 6168.87 | < 0.01 | 3.47 | 2.59 |
| 9959.63 | < 0.01 | 1.31 | 2.86 |
| 11138.33 | < 0.01 | 1.22 | 0.91 |
| 12537.43 | < 0.01 | 0.94 | 0.69 |
| 2813.75 | < 0.01 | 3.78 | 7.16 |
| 10502.28 | < 0.01 | 2.28 | 1.24 |
| 3322.95 | < 0.01 | 4.65 | 3.59 |
| 3039.79 | < 0.01 | 3.54 | 5.32 |
| 4962.96 | < 0.01 | 13.03 | 37.17 |
| 4983.85 | < 0.01 | 5.33 | 13.06 |
| 2830.6 | < 0.01 | 4.01 | 7.25 |
| 4333.38 | < 0.01 | 3.02 | 5.16 |
| 5168.85 | < 0.01 | 2.93 | 4.54 |
| 7639.3 | < 0.01 | 1.61 | 1.32 |
| 4540.4 | < 0.01 | 2.68 | 3.66 |
| 3440 | < 0.01 | 4.27 | 3.42 |
| 17451.23 | < 0.01 | 0.58 | 0.42 |
| 20689.06 | < 0.01 | 0.46 | 0.35 |
| 4746.93 | < 0.05 | 3.44 | 6.67 |
| 4278.44 | < 0.05 | 3.08 | 4.01 |
| 10579.88 | < 0.05 | 1.36 | 1.06 |
| 24759.57 | < 0.05 | 0.44 | 0.32 |
| 20975.94 | < 0.05 | 0.46 | 0.35 |
| 11099.96 | < 0.05 | 1.03 | 0.86 |
| 5022.15 | < 0.05 | 3.7 | 6.37 |
| 4836.74 | < 0.05 | 5.02 | 3.42 |
| 4919.44 | < 0.05 | 2.86 | 3.53 |
| 11170.36 | < 0.05 | 1.05 | 0.89 |
| 7003.99 | < 0.05 | 2.21 | 1.94 |
| 11055.96 | < 0.05 | 1.14 | 0.97 |
| 14403.69 | < 0.05 | 0.8 | 0.65 |
| 5060.07 | < 0.05 | 2.93 | 3.96 |
| 20734.74 | < 0.05 | 0.43 | 0.35 |

**Significant *m/z*-species from comparison wild type vs IPMN**

| **Mass** | **PWKW** | **Ave WT** | **Ave IPMN** |
| --- | --- | --- | --- |
| 3530.62 | < 0.001 | 3.7 | 4.92 |
| 6604.28 | < 0.001 | 3.66 | 2.44 |
| 6645.81 | < 0.001 | 19.76 | 6.04 |
| 4334.02 | < 0.001 | 3.06 | 5.06 |
| 4239.16 | < 0.001 | 3.21 | 4.4 |
| 4165.67 | < 0.001 | 3.12 | 4.23 |
| 6274.04 | < 0.001 | 10.67 | 4.63 |
| 6222.09 | < 0.001 | 9.03 | 4.53 |
| 6683.78 | < 0.001 | 5.23 | 2.42 |
| 4541.08 | < 0.001 | 2.79 | 3.72 |
| 3513.67 | < 0.001 | 3.87 | 4.66 |
| 14001.34 | < 0.001 | 1.96 | 1.11 |
| 6167.9 | < 0.001 | 3.95 | 2.87 |
| 4277.83 | < 0.001 | 3.07 | 4.1 |
| 3492.48 | < 0.001 | 4.82 | 8.31 |
| 6118.91 | < 0.001 | 3.66 | 2.57 |
| 3001.32 | < 0.01 | 3.86 | 4.99 |
| 6311.13 | < 0.01 | 3.75 | 2.69 |
| 11137.79 | < 0.01 | 1.22 | 0.92 |
| 2790.78 | < 0.01 | 5.81 | 17.64 |
| 10501.63 | < 0.01 | 2.26 | 1.29 |
| 6141.25 | < 0.01 | 2.8 | 2.25 |
| 2829.5 | < 0.01 | 4 | 5.45 |
| 14409.62 | < 0.01 | 0.78 | 0.6 |
| 11056.09 | < 0.01 | 1.06 | 0.85 |
| 17456.17 | < 0.01 | 0.5 | 0.38 |
| 2813 | < 0.01 | 3.8 | 5.3 |
| 3058.99 | < 0.01 | 3.75 | 4.61 |
| 11102.19 | < 0.01 | 1 | 0.82 |
| 21788.64 | < 0.01 | 0.43 | 0.33 |
| 2928.46 | < 0.01 | 3.72 | 4.63 |
| 6570.16 | < 0.01 | 2.86 | 2.26 |
| 12535.09 | < 0.01 | 0.94 | 0.73 |
| 6850.56 | < 0.05 | 2.33 | 1.99 |
| 10639.75 | < 0.05 | 1.08 | 0.86 |
| 6722.7 | < 0.05 | 2.45 | 1.84 |
| 3423.34 | < 0.05 | 3.91 | 4.44 |
| 11035.48 | < 0.05 | 0.97 | 0.82 |
| 12060.97 | < 0.05 | 0.85 | 0.71 |
| 11213.27 | < 0.05 | 1.02 | 0.86 |
| 3454.89 | < 0.05 | 7.93 | 5.93 |
| 4983.7 | < 0.05 | 5.24 | 8.12 |
| 4746.48 | < 0.05 | 3.42 | 4.38 |
| 5764.58 | < 0.05 | 2.36 | 3.2 |
| 11001.87 | < 0.05 | 1.03 | 0.91 |
| 15186.74 | < 0.05 | 0.65 | 0.55 |

**Significant *m/z*-species from comparison wild type vs PanIN**

| **Mass** | **PWKW** | **Ave WT** | **Ave PanIN** |
| --- | --- | --- | --- |
| 6645.57 | < 0.000001 | 19.88 | 3.88 |
| 2812.81 | < 0.000001 | 3.78 | 7.77 |
| 6683.46 | < 0.000001 | 5.32 | 2.24 |
| 6189.01 | < 0.000001 | 3.91 | 2.38 |
| 6604.32 | < 0.000001 | 3.68 | 2.23 |
| 6310.84 | < 0.000001 | 3.83 | 2.41 |
| 2790.74 | < 0.000001 | 5.83 | 27.36 |
| 6273.78 | < 0.000001 | 10.71 | 3.56 |
| 6222.05 | < 0.000001 | 9.06 | 3.4 |
| 4334.21 | < 0.000001 | 3.02 | 7.21 |
| 2829.86 | < 0.00001 | 4 | 7.68 |
| 6165.7 | < 0.00001 | 3.45 | 2.29 |
| 4165.53 | < 0.00001 | 3.1 | 4.36 |
| 6138.83 | < 0.00001 | 2.8 | 2.09 |
| 6118.58 | < 0.00001 | 3.63 | 2.4 |
| 6573.03 | < 0.00001 | 2.67 | 2.04 |
| 11138.83 | < 0.0001 | 1.23 | 0.88 |
| 4319.57 | < 0.0001 | 2.93 | 4.14 |
| 3789.22 | < 0.0001 | 3.26 | 5.67 |
| 3533.02 | < 0.0001 | 3.71 | 4.93 |
| 11080.49 | < 0.0001 | 0.95 | 0.73 |
| 7418.34 | < 0.001 | 1.83 | 3.71 |
| 4277.76 | < 0.001 | 3.13 | 4.13 |
| 14000.87 | < 0.001 | 1.98 | 1.06 |
| 10501.7 | < 0.001 | 2.27 | 1.24 |
| 12538.25 | < 0.001 | 0.93 | 0.71 |
| 4542.17 | < 0.001 | 2.73 | 3.85 |
| 3576.52 | < 0.001 | 3.36 | 5.28 |
| 3000.49 | < 0.001 | 3.88 | 5.01 |
| 11102.36 | < 0.001 | 0.98 | 0.78 |
| 6721.28 | < 0.001 | 2.61 | 1.97 |
| 7219.48 | < 0.001 | 1.94 | 3.69 |
| 15695.28 | < 0.001 | 0.62 | 0.46 |
| 6975.7 | < 0.001 | 2.22 | 1.91 |
| 5341.74 | < 0.001 | 2.31 | 2.92 |
| 14395.28 | < 0.001 | 0.69 | 0.52 |
| 11211.78 | < 0.01 | 0.96 | 0.77 |
| 15191.66 | < 0.01 | 0.65 | 0.52 |
| 3454.71 | < 0.01 | 7.89 | 5.09 |
| 6549.13 | < 0.01 | 3.01 | 2.52 |
| 12506.55 | < 0.01 | 0.79 | 0.61 |
| 11056.61 | < 0.01 | 1.13 | 0.94 |
| 10394.43 | < 0.01 | 1.08 | 0.88 |
| 14973.3 | < 0.01 | 0.79 | 0.62 |
| 10366.15 | < 0.01 | 1.13 | 0.93 |
| 14445.38 | < 0.01 | 0.74 | 0.56 |
| 3473.73 | < 0.01 | 4.49 | 6.59 |
| 3428.85 | < 0.01 | 3.93 | 4.75 |
| 11004.75 | < 0.01 | 1.01 | 0.86 |
| 14615.2 | < 0.01 | 0.63 | 0.49 |
| 14798.83 | < 0.01 | 0.63 | 0.5 |
| 14314.19 | < 0.01 | 0.73 | 0.56 |
| 15614.77 | < 0.01 | 0.63 | 0.49 |
| 4046.56 | < 0.01 | 3.32 | 4.23 |
| 14743.51 | < 0.01 | 0.61 | 0.49 |
| 14845.83 | < 0.01 | 0.58 | 0.46 |
| 17446.51 | < 0.01 | 0.53 | 0.41 |
| 4836.38 | < 0.01 | 5.02 | 3.47 |
| 17012.19 | < 0.01 | 0.49 | 0.39 |
| 22155.52 | < 0.01 | 0.43 | 0.36 |
| 5078.23 | < 0.01 | 2.72 | 3.2 |
| 10893.48 | < 0.01 | 0.96 | 0.83 |
| 5059.92 | < 0.05 | 2.92 | 3.46 |
| 11537.97 | < 0.05 | 1.01 | 0.85 |
| 8943.89 | < 0.05 | 1.34 | 1.18 |
| 13642.93 | < 0.05 | 0.63 | 0.53 |
| 16152.94 | < 0.05 | 0.54 | 0.44 |
| 3513.89 | < 0.05 | 3.78 | 4.57 |
| 6850.39 | < 0.05 | 2.33 | 2.07 |
| 14771.67 | < 0.05 | 0.64 | 0.51 |
| 17160.36 | < 0.05 | 0.49 | 0.4 |
| 14931.5 | < 0.05 | 0.65 | 0.51 |
| 10976.4 | < 0.05 | 0.95 | 0.83 |
| 15430.26 | < 0.05 | 0.61 | 0.51 |
| 3949.53 | < 0.05 | 4.79 | 6.87 |
| 13697.54 | < 0.05 | 0.73 | 0.63 |
| 15272.36 | < 0.05 | 0.65 | 0.53 |
| 13615.66 | < 0.05 | 0.67 | 0.59 |
| 20735.67 | < 0.05 | 0.42 | 0.34 |
| 18586.18 | < 0.05 | 0.45 | 0.38 |
| 5168.06 | < 0.05 | 2.92 | 3.4 |
| 16980.38 | < 0.05 | 0.49 | 0.41 |
| 21785.11 | < 0.05 | 0.49 | 0.42 |
| 22075.49 | < 0.05 | 0.43 | 0.35 |
| 4983.43 | < 0.05 | 5.29 | 7.71 |
| 5021.31 | < 0.05 | 3.7 | 4.63 |
| 11596.16 | < 0.05 | 1.05 | 0.92 |
| 18667.48 | < 0.05 | 0.48 | 0.4 |
| 20684.92 | < 0.05 | 0.46 | 0.38 |
| 13842.09 | < 0.05 | 0.73 | 0.61 |
| 10935.39 | < 0.05 | 1.16 | 1.02 |
| 10872.07 | < 0.05 | 0.9 | 0.82 |

**Significant *m/z*-species from comparison wild type vs Lesion (PanIN and IPMN)**

| **Mass** | **PWKW** | **Ave WT** | **Ave Lesion** |
| --- | --- | --- | --- |
| 6604.52 | < 0.000001 | 3.65 | 2.32 |
| 6645.95 | < 0.00001 | 19.72 | 4.91 |
| 6222.22 | < 0.00001 | 9.02 | 3.96 |
| 6684.08 | < 0.00001 | 5.25 | 2.43 |
| 4334.15 | < 0.00001 | 3.02 | 5.91 |
| 6189.79 | < 0.00001 | 3.91 | 2.57 |
| 6274.22 | < 0.00001 | 10.67 | 4.24 |
| 6140.21 | < 0.00001 | 2.81 | 2.18 |
| 2813.03 | < 0.00001 | 3.74 | 6.76 |
| 4237.05 | < 0.00001 | 3.21 | 4.16 |
| 2790.95 | < 0.0001 | 5.83 | 22.79 |
| 4165 | < 0.0001 | 3.11 | 4.09 |
| 3531.69 | < 0.0001 | 3.72 | 5 |
| 6166.41 | < 0.0001 | 3.43 | 2.48 |
| 3576.44 | < 0.0001 | 3.35 | 4.85 |
| 6311.16 | < 0.0001 | 3.73 | 2.56 |
| 6118.92 | < 0.0001 | 3.64 | 2.58 |
| 4541.56 | < 0.0001 | 2.74 | 3.78 |
| 14001.39 | < 0.0001 | 1.96 | 1.12 |
| 3789.62 | < 0.0001 | 3.26 | 5.03 |
| 4319.84 | < 0.0001 | 2.82 | 3.79 |
| 2829.68 | < 0.0001 | 4.01 | 6.92 |
| 3000.78 | < 0.0001 | 4 | 5.02 |
| 4278.29 | < 0.0001 | 3.16 | 4.08 |
| 10502.19 | < 0.001 | 2.27 | 1.29 |
| 11137.94 | < 0.001 | 1.22 | 0.92 |
| 11079.85 | < 0.001 | 0.94 | 0.75 |
| 6572.86 | < 0.001 | 2.66 | 2.17 |
| 6721.56 | < 0.001 | 2.8 | 2.07 |
| 14408.88 | < 0.001 | 0.73 | 0.56 |
| 14207.26 | < 0.001 | 1.02 | 0.78 |
| 3454.83 | < 0.01 | 7.88 | 5.22 |
| 3513.74 | < 0.01 | 3.8 | 4.55 |
| 10576.87 | < 0.01 | 1.29 | 0.96 |
| 7418.57 | < 0.01 | 1.89 | 3.16 |
| 12537.58 | < 0.01 | 0.98 | 0.77 |
| 11101.77 | < 0.01 | 1 | 0.82 |
| 17455.45 | < 0.01 | 0.59 | 0.45 |
| 7219.52 | < 0.01 | 1.99 | 3.15 |
| 11056.67 | < 0.01 | 1.12 | 0.93 |
| 13161.19 | < 0.01 | 0.68 | 0.56 |
| 10391.13 | < 0.01 | 1.09 | 0.91 |
| 11211.99 | < 0.01 | 1.01 | 0.84 |
| 14746.9 | < 0.01 | 0.64 | 0.52 |
| 15618.42 | < 0.01 | 0.65 | 0.52 |
| 15654.28 | < 0.01 | 0.6 | 0.48 |
| 6975.77 | < 0.01 | 2.21 | 1.97 |
| 15186.81 | < 0.01 | 0.69 | 0.58 |
| 3493.38 | < 0.01 | 4.81 | 8.03 |
| 15696.38 | < 0.01 | 0.66 | 0.52 |
| 8944.05 | < 0.01 | 1.31 | 1.18 |
| 14791.57 | < 0.01 | 0.74 | 0.61 |
| 11004.05 | < 0.01 | 0.97 | 0.84 |
| 3425.45 | < 0.01 | 3.95 | 4.55 |
| 14975.31 | < 0.01 | 0.73 | 0.59 |
| 10363.87 | < 0.01 | 1.13 | 0.99 |
| 24841.63 | < 0.01 | 0.42 | 0.35 |
| 11236.49 | < 0.05 | 0.9 | 0.79 |
| 4046.81 | < 0.05 | 3.19 | 3.88 |
| 5168.19 | < 0.05 | 2.95 | 3.45 |
| 3473.85 | < 0.05 | 4.49 | 5.68 |
| 2850.6 | < 0.05 | 3.73 | 4.55 |
| 20668.61 | < 0.05 | 0.44 | 0.37 |
| 6850.77 | < 0.05 | 2.32 | 2.05 |
| 13359.43 | < 0.05 | 0.73 | 0.64 |
| 5078.18 | < 0.05 | 2.72 | 3.15 |
| 13236.04 | < 0.05 | 0.73 | 0.64 |
| 12236.23 | < 0.05 | 0.87 | 0.78 |
| 13637.89 | < 0.05 | 0.63 | 0.55 |
| 21880.76 | < 0.05 | 0.54 | 0.46 |
| 5060.18 | < 0.05 | 2.92 | 3.42 |
| 20962.1 | < 0.05 | 0.43 | 0.36 |
| 4983.54 | < 0.05 | 5.27 | 7.47 |
| 13405.54 | < 0.05 | 0.75 | 0.66 |
| 4937.09 | < 0.05 | 3.42 | 4.21 |
| 8363.86 | < 0.05 | 1.38 | 1.27 |
